# Supplementary material for: Polyketide synthase gene clusters in the ericoid mycorrhizal fungus Oidiodendron maius: genome-wide prediction, expression profiling, and functional characterization of knockout mutants
Source: Front Plant Sci. 2026 Sep 16;17:1924430. doi: 10.3389/fpls.2026.1924430 (PMC13623903; doi:10.3389/fpls.2026.1924430)
Supplement: Supplementary file 1 [file SupplementaryFile1.zip › Supplementary Materials/Supplementary Folder 1.zip!/Oidma1_AssemblyScaffolds_Repeatmasked/knownclusterblast/cluster33/jgi.p_Oidma1_58000_mibig_hits.html]

| MIBiG Protein | Description | MIBiG Cluster | MiBiG Product | % ID | % Coverage | BLAST Score | E-value |
| --- | --- | --- | --- | --- | --- | --- | --- |
| ABB76806.1 | polyketide\_synthase\_2 | BGC0000155\_c2 | T-toxin\_biosynthetic\_gene\_cluster | 30 | 101.486486486 | 829 | 9.4e-240 |
| AAB08104.3 | polyketide\_synthase | BGC0000155\_c3 | T-toxin\_biosynthetic\_gene\_cluster | 39 | 58.7837837838 | 809 | 7.7e-234 |
| EWG54266.1 | hypothetical\_protein | BGC0001190 | Fusaric\_acid\_biosynthetic\_gene\_cluster | 37 | 59.1441441441 | 799 | 1e-230 |
| ANF07288.1 | hrPKS | BGC0001385 | Byssochlamic\_acid\_/\_agnestadrides\_biosynthetic\_gene\_cluster | 40 | 55.8108108108 | 796 | 6.7e-230 |
| BAD83684.1 | PKSN\_polyketide\_synthase\_for\_alternapyrone\_biosynthesis | BGC0000012 | Alternapyrone\_biosynthetic\_gene\_cluster | 36 | 59.3243243243 | 738 | 2.2e-212 |
| CAP95405.1 |  | BGC0001404 | Sorbicillin\_biosynthetic\_gene\_cluster | 36 | 59.0990990991 | 733 | 9.2e-211 |
| KGO40478.1 | Acyl\_transferase/acyl\_hydrolase/lysophospholipase | BGC0001205 | Communesin\_biosynthetic\_gene\_cluster | 36 | 57.7027027027 | 713 | 7.5e-205 |
| BAV32159.1 | polyketide\_synthase | BGC0001373 | Sordarin\_biosynthetic\_gene\_cluster | 34 | 60.2702702703 | 711 | 3.7e-204 |
| AMY15057.1 | tetraketide\_synthase\_MF-SQTKS | BGC0001339 | Squalestatin\_S1\_biosynthetic\_gene\_cluster | 35 | 60.3153153153 | 708 | 2.4e-203 |
| EHA28244.1 | hypothetical\_protein | BGC0001143 | Azanigerone\_biosynthetic\_gene\_cluster | 34 | 61.036036036 | 703 | 1e-201 |
| AHV78245.1 | LasS1 | BGC0001245 | Lasiodiplodin\_biosynthetic\_gene\_cluster | 36 | 57.5675675676 | 699 | 1.5e-200 |
| CBF87072.1 | polyketide\_synthase,\_putative\_(Eurofung) | BGC0001290 | Emericellamide\_A\_/\_emericellamide\_B\_biosynthetic\_gene\_cluster | 34 | 60.7657657658 | 699 | 1.5e-200 |
| AGC95324.1 | CurS1 | BGC0000045 | Dehydrocurvularin\_biosynthetic\_gene\_cluster | 35 | 58.6036036036 | 693 | 1.1e-198 |
| ACD39767.1 | reducing\_polyketide\_synthase | BGC0000077 | Hypothemycin\_biosynthetic\_gene\_cluster | 36 | 57.8828828829 | 685 | 2.9e-196 |
| ACD39758.1 | reducing\_polyketide\_synthase | BGC0000076 | Hypothemycin\_biosynthetic\_gene\_cluster | 36 | 57.8828828829 | 685 | 2.9e-196 |
| AHV78252.1 | ResS1 | BGC0001246 | Trans-resorcylide\_biosynthetic\_gene\_cluster | 34 | 66.0810810811 | 684 | 4.9e-196 |
| BAC20566.1 | polyketide\_synthase | BGC0000039 | Compactin\_biosynthetic\_gene\_cluster | 34 | 59.4594594595 | 675 | 3e-193 |
| ABA02240.1 | polyketide\_synthase | BGC0000098 | Monacolin\_K\_biosynthetic\_gene\_cluster | 34 | 58.0630630631 | 671 | 4.3e-192 |
| AAD34559.1 | polyketide\_synthase | BGC0000089 | Lovastatin\_biosynthetic\_gene\_cluster | 34 | 59.2342342342 | 663 | 1.2e-189 |
| ACD39774.1 | reducing\_polyketide\_synthase | BGC0000134 | Radicicol\_biosynthetic\_gene\_cluster | 35 | 57.4774774775 | 658 | 2.2e-188 |
| ABB90283.1 | polyketide\_synthase | BGC0001057 | Zearalenone\_biosynthetic\_gene\_cluster | 34 | 56.8918918919 | 657 | 4.9e-188 |
| ACB12550.1 | Fum1 | BGC0000063 | Fumonisin\_biosynthetic\_gene\_cluster | 34 | 56.4414414414 | 620 | 8.7e-177 |
| AAD43562.2 | Fum1p | BGC0000062 | Fumonisin\_biosynthetic\_gene\_cluster | 34 | 56.6216216216 | 618 | 4.3e-176 |
| AKL78824.1 | GLPKS3 | BGC0001187 | Xenolozoyenone\_biosynthetic\_gene\_cluster | 34 | 59.3243243243 | 610 | 9e-174 |
| EAA65604.1 | hypothetical\_protein | BGC0000022 | Asperfuranone\_biosynthetic\_gene\_cluster | 33 | 60.8558558559 | 608 | 3.4e-173 |
| AAY28225.1 | HbmAI | BGC0000074 | Herbimycin\_biosynthetic\_gene\_cluster | 28 | 93.3783783784 | 604 | 6.4e-172 |
| AIA58899.1 | HRPKS | BGC0001141 | Brefeldin\_biosynthetic\_gene\_cluster | 33 | 55.8108108108 | 599 | 2.1e-170 |
| EAL85129.1 | polyketide\_synthase,\_putative | BGC0001067 | Fumagillin\_biosynthetic\_gene\_cluster | 33 | 58.5585585586 | 597 | 7.8e-170 |
| AAT28740.1 | FUSS | BGC0000064 | Fusarin\_biosynthetic\_gene\_cluster | 33 | 55.2252252252 | 574 | 4.2e-163 |
| EPE34340.1 | Thiolase-like\_protein | BGC0001035 | Pneumocandin\_biosynthetic\_gene\_cluster | 32 | 56.8468468468 | 572 | 2.7e-162 |
| CAO91861.1 | putative\_PKS-NRPS\_protein | BGC0000968 | Chaetoglobosin\_biosynthetic\_gene\_cluster | 32 | 60.0 | 571 | 4.6e-162 |
| ACS68554.1 | hybrid\_PKS-NRPS\_protein | BGC0001026 | NG-391\_biosynthetic\_gene\_cluster | 33 | 55.7207207207 | 566 | 1.5e-160 |
| ADN43685.1 | DmbS | BGC0001136 | Desmethylbassianin\_biosynthetic\_gene\_cluster | 33 | 57.7927927928 | 564 | 4.3e-160 |
| CAL69597.1 | nonribosomal\_peptide\_synthase | BGC0001049 | Tenellin\_biosynthetic\_gene\_cluster | 32 | 61.4414414414 | 563 | 9.6e-160 |
| ACZ57548.1 | polyketide\_synthase | BGC0000046 | Depudecin\_biosynthetic\_gene\_cluster | 32 | 55.0 | 563 | 1.3e-159 |
| AFP73394.1 | FusA | BGC0001268 | Fusarin\_biosynthetic\_gene\_cluster | 34 | 55.1801801802 | 561 | 6.2e-159 |
| CBF80487.1 | hybrid\_PKS-NRPS\_(Eurofung) | BGC0000959 | Aspyridone\_biosynthetic\_gene\_cluster | 32 | 61.981981982 | 552 | 2.9e-156 |
| BAN19720.1 | polyketide\_synthase | BGC0001252 | ACR-toxin\_biosynthetic\_gene\_cluster | 33 | 56.0810810811 | 549 | 1.4e-155 |
| ABA02239.1 | polyketide\_synthase | BGC0000098 | Monacolin\_K\_biosynthetic\_gene\_cluster | 32 | 56.9369369369 | 547 | 5.5e-155 |
| AKC54422.1 | fumosorinone\_biosynthesis\_polyketide\_synthase | BGC0001218 | Fumosorinone\_biosynthetic\_gene\_cluster | 32 | 61.5315315315 | 546 | 1.2e-154 |
| BAJ09789.1 | polyketide\_synthase | BGC0000146 | Solanapyrone\_biosynthetic\_gene\_cluster | 30 | 59.6396396396 | 542 | 3e-153 |
| EAL85113.2 | hybrid\_PKS-NRPS\_enzyme,\_putative | BGC0001037 | Pseurotin\_A\_biosynthetic\_gene\_cluster | 35 | 49.7747747748 | 540 | 6.7e-153 |
| BAJ14522.1 | polyketide\_synthase | BGC0001253\_c4 | ACT-Toxin\_I\_biosynthetic\_gene\_cluster | 35 | 48.6936936937 | 539 | 1.5e-152 |
| BAD97694.1 | Aft9-1 | BGC0000003 | AF-toxin\_biosynthetic\_gene\_cluster | 32 | 55.3603603604 | 539 | 2.5e-152 |
| EAL89230.2 | LovB-like\_polyketide\_synthase,\_putative | BGC0000129 | Pyripyropene\_biosynthetic\_gene\_cluster | 32 | 55.045045045 | 533 | 1.1e-150 |
| BAC20564.1 | polyketide\_synthase | BGC0000039 | Compactin\_biosynthetic\_gene\_cluster | 32 | 56.6666666667 | 529 | 2.6e-149 |
| EAW09117.1 | hybrid\_NRPS/PKS\_enzyme,\_putative | BGC0000983 | Cytochalasin\_biosynthetic\_gene\_cluster | 31 | 59.5495495495 | 528 | 4.5e-149 |
| CCT72377.1 | probable\_polyketide\_synthase | BGC0001305 | Fujikurins\_biosynthetic\_gene\_cluster | 34 | 50.0 | 525 | 2.2e-148 |
| EAU38971.1 | hypothetical\_protein | BGC0001122 | Isoflavipucine\_biosynthetic\_gene\_cluster | 35 | 48.5585585586 | 524 | 4.9e-148 |
| ctg1\_orf0002 |  | BGC0001068 | Pyripyropene\_A\_biosynthetic\_gene\_cluster | 34 | 48.9189189189 | 524 | 6.5e-148 |
| AAV66110.2 | fusaridione\_A\_synthetase | BGC0000992 | Fusaridione\_A\_biosynthetic\_gene\_cluster | 32 | 56.7117117117 | 516 | 1.3e-145 |
| EAQ86385.1 | hypothetical\_protein | BGC0001405 | Chaetoviridin\_/\_chaetomugilin\_biosynthetic\_gene\_cluster | 41 | 36.1261261261 | 510 | 9.7e-144 |
| AGO86662.1 | equisetin\_synthetase | BGC0001255 | Equisetin\_biosynthetic\_gene\_cluster | 34 | 49.009009009 | 506 | 1.4e-142 |
| BAQ25466.1 | polyketide\_synthase | BGC0001280 | Betaenone\_C\_/\_betaenone\_A\_biosynthetic\_gene\_cluster | 35 | 47.2072072072 | 504 | 6.9e-142 |
| AMY15068.1 | hexaketide\_synthase\_MF-SQHKS | BGC0001339 | Squalestatin\_S1\_biosynthetic\_gene\_cluster | 30 | 53.6936936937 | 495 | 3.2e-139 |
| CBX87032.1 | BcBOA9\_protein | BGC0001243\_c3 | Botryenalol\_/\_botrydial\_/\_dihydrobotrydial\_/\_botryendial\_/ | 30 | 57.027027027 | 494 | 7.2e-139 |
| BAK26562.1 | PKS-NRPS\_hybrid | BGC0000977 | Cyclopiazonic\_acid\_biosynthetic\_gene\_cluster | 34 | 47.1171171171 | 489 | 1.8e-137 |
| CAG28678.1 | polyketide\_synthase | BGC0001023 | Myxochromide\_biosynthetic\_gene\_cluster | 31 | 53.2432432432 | 473 | 1.7e-132 |
| CAQ43078.1 | polyketide\_synthase | BGC0000970 | Chondrochloren\_biosynthetic\_gene\_cluster | 31 | 55.5855855856 | 472 | 3.8e-132 |
| AAK57189.1 | MxaE | BGC0001022 | Myxalamid\_biosynthetic\_gene\_cluster | 30 | 54.6846846847 | 471 | 5e-132 |
| CAD19091.1 | StiG\_protein | BGC0000153 | Stigmatellin\_biosynthetic\_gene\_cluster | 31 | 54.0540540541 | 468 | 5.5e-131 |
| CAQ18830.1 | polyketide\_synthase | BGC0000954 | Ajudazol\_biosynthetic\_gene\_cluster | 30 | 56.2162162162 | 468 | 5.5e-131 |
| AAT70105.1 | CurJ | BGC0001165 | Curacin\_biosynthetic\_gene\_cluster | 30 | 55.9459459459 | 463 | 1.4e-129 |
| AEE88280.1 | CurJ | BGC0000976 | Curacin\_biosynthetic\_gene\_cluster | 30 | 55.9459459459 | 463 | 1.4e-129 |
| AAF19810.1 | MtaB | BGC0001024 | Myxothiazol\_biosynthetic\_gene\_cluster | 30 | 55.6306306306 | 462 | 3.9e-129 |
| CAJ46689.1 | polyketide\_synthase | BGC0000969 | Chondramide\_biosynthetic\_gene\_cluster | 31 | 54.8648648649 | 461 | 5.1e-129 |
| CAJ46690.1 | polyketide\_synthase | BGC0000969 | Chondramide\_biosynthetic\_gene\_cluster | 31 | 54.6396396396 | 460 | 8.8e-129 |
| EHA19289.1 | hypothetical\_protein | BGC0001124 | Pyranonigrin\_E\_biosynthetic\_gene\_cluster | 33 | 40.0 | 456 | 1.3e-127 |
| AGC45620.1 | polyketide\_synthase | BGC0001394 | Phenalamide\_biosynthetic\_gene\_cluster | 33 | 42.7027027027 | 450 | 9.1e-126 |
| BAG17643.1 | putative\_NRPS-type-I\_PKS\_fusion\_protein | BGC0001043 | SGR\_PTMs\_biosynthetic\_gene\_cluster | 30 | 54.4594594595 | 450 | 9.1e-126 |
| ABX60162.1 | polyketide\_synthase | BGC0000978 | Cylindrospermopsin\_biosynthetic\_gene\_cluster | 30 | 55.1351351351 | 450 | 9.1e-126 |
| AAS98783.1 | JamL | BGC0001001 | Jamaicamide\_biosynthetic\_gene\_cluster | 29 | 55.8108108108 | 450 | 1.2e-125 |
| ADF88280.1 | polyketide\_synthase | BGC0000981 | Cylindrospermopsin\_biosynthetic\_gene\_cluster | 30 | 55.1351351351 | 447 | 1e-124 |
| ACR33078.1 | polyketide\_synthase | BGC0000017 | Anatoxin\_biosynthetic\_gene\_cluster | 30 | 54.7297297297 | 447 | 1e-124 |
| ABL86391.1 | hybrid\_polyketide\_synthase\_and\_nonribosomal\_peptide\_synthetase | BGC0000999 | Heat-stable\_antifungal\_factor\_biosynthetic\_gene\_cluster | 31 | 54.0990990991 | 446 | 1.3e-124 |
| CBD77732.1 | polyketide\_synthase | BGC0000974 | Crocacin\_biosynthetic\_gene\_cluster | 32 | 43.018018018 | 445 | 3.8e-124 |
| AEE88278.1 | CurL | BGC0000976 | Curacin\_biosynthetic\_gene\_cluster | 33 | 43.018018018 | 443 | 1.4e-123 |
| AAT70107.1 | CurL | BGC0001165 | Curacin\_biosynthetic\_gene\_cluster | 33 | 43.018018018 | 443 | 1.4e-123 |
| XP\_001220460.1 | hypothetical\_protein | BGC0001182 | Chaetoglobosins\_biosynthetic\_gene\_cluster | 31 | 49.7297297297 | 443 | 1.9e-123 |
| CAQ18834.1 | polyketide\_synthase | BGC0000954 | Ajudazol\_biosynthetic\_gene\_cluster | 33 | 42.6576576577 | 439 | 2.7e-122 |
| CAO98850.1 | polyketide\_synthase\_AufG | BGC0000023 | Aurafuron\_biosynthetic\_gene\_cluster | 30 | 55.6306306306 | 438 | 4.7e-122 |
| CAQ18829.1 | polyketide\_synthase | BGC0000954 | Ajudazol\_biosynthetic\_gene\_cluster | 30 | 55.045045045 | 438 | 4.7e-122 |
| ADC45538.1 | modular\_polyketide\_synthase | BGC0000093 | Meilingmycin\_biosynthetic\_gene\_cluster | 32 | 49.3243243243 | 437 | 8e-122 |
| AAF19813.1 | MtaE | BGC0001024 | Myxothiazol\_biosynthetic\_gene\_cluster | 32 | 43.1531531532 | 436 | 1.4e-121 |
| EAU29808.1 | hypothetical\_protein | BGC0001400 | Citreoviridin\_biosynthetic\_gene\_cluster | 32 | 44.4594594595 | 436 | 1.8e-121 |
| CCE88381.1 | polyketide\_synthase | BGC0001034 | Pellasoren\_biosynthetic\_gene\_cluster | 30 | 55.1801801802 | 435 | 5.2e-121 |
| CBD77738.1 | polyketide\_synthase | BGC0000974 | Crocacin\_biosynthetic\_gene\_cluster | 32 | 42.7027027027 | 430 | 9.7e-120 |
| AAK57187.1 | MxaC | BGC0001022 | Myxalamid\_biosynthetic\_gene\_cluster | 34 | 42.8828828829 | 427 | 8.2e-119 |
| AEE88279.1 | CurK | BGC0000976 | Curacin\_biosynthetic\_gene\_cluster | 28 | 56.1261261261 | 427 | 8.2e-119 |
| AAT70106.1 | CurK | BGC0001165 | Curacin\_biosynthetic\_gene\_cluster | 28 | 56.1261261261 | 427 | 8.2e-119 |
| CBD77748.1 | polyketide\_synthase | BGC0000974 | Crocacin\_biosynthetic\_gene\_cluster | 29 | 54.8648648649 | 425 | 5.3e-118 |
| ALD82524.1 | polyketide\_synthase | BGC0001212 | Nannocystin\_biosynthetic\_gene\_cluster | 30 | 55.4954954955 | 423 | 1.2e-117 |
| ADZ24997.1 | polyketide\_synthase | BGC0000380 | Leupyrrin\_biosynthetic\_gene\_cluster | 28 | 67.2522522523 | 423 | 1.6e-117 |
| AEE88281.1 | CurI | BGC0000976 | Curacin\_biosynthetic\_gene\_cluster | 32 | 43.4684684685 | 423 | 2e-117 |
| AAT70104.1 | CurI | BGC0001165 | Curacin\_biosynthetic\_gene\_cluster | 32 | 43.4684684685 | 423 | 2e-117 |
| ALD82522.1 | polyketide\_synthase | BGC0001212 | Nannocystin\_biosynthetic\_gene\_cluster | 30 | 55.5405405405 | 422 | 3.5e-117 |
| AAW03328.1 | CtaE | BGC0000982 | Cystothiazole\_A\_biosynthetic\_gene\_cluster | 32 | 42.8828828829 | 419 | 2.2e-116 |
| CAD19088.1 | StiD\_protein | BGC0000153 | Stigmatellin\_biosynthetic\_gene\_cluster | 32 | 42.7927927928 | 419 | 2.9e-116 |
| AIW82282.1 | PuwE | BGC0001125 | Puwainaphycins\_biosynthetic\_gene\_cluster | 32 | 42.8828828829 | 418 | 3.8e-116 |
| AGY30677.1 | Ann5 | BGC0001298 | 4-Z-annimycin\_biosynthetic\_gene\_cluster | 32 | 46.6666666667 | 418 | 5e-116 |
| AMB48442.1 | polyketide\_synthase | BGC0001357 | Carbamidocyclophane\_biosynthetic\_gene\_cluster | 32 | 43.2882882883 | 417 | 8.5e-116 |
| ACR50774.1 | polyketide\_synthase | BGC0000163 | Tetronasin\_biosynthetic\_gene\_cluster | 30 | 49.6846846847 | 417 | 1.1e-115 |
| ADF88276.1 | polyketide\_synthase | BGC0000981 | Cylindrospermopsin\_biosynthetic\_gene\_cluster | 30 | 53.963963964 | 416 | 1.5e-115 |
| AAS98782.1 | JamK | BGC0001001 | Jamaicamide\_biosynthetic\_gene\_cluster | 31 | 44.8648648649 | 416 | 1.9e-115 |
| CAQ18833.1 | polyketide\_synthase | BGC0000954 | Ajudazol\_biosynthetic\_gene\_cluster | 33 | 42.5675675676 | 416 | 2.5e-115 |
| AAF26921.1 | polyketide\_synthase | BGC0000988 | Epothilone\_biosynthetic\_gene\_cluster | 29 | 54.2342342342 | 415 | 3.2e-115 |
| CAO98879.1 | polyketide\_synthase\_AufD | BGC0000023 | Aurafuron\_biosynthetic\_gene\_cluster | 31 | 42.8828828829 | 415 | 4.2e-115 |
| CAD19087.1 | StiC\_protein | BGC0000153 | Stigmatellin\_biosynthetic\_gene\_cluster | 29 | 56.1711711712 | 415 | 5.5e-115 |
| AIT55260.1 | GulC | BGC0000072 | Gulmirecin\_biosynthetic\_gene\_cluster | 28 | 56.981981982 | 415 | 5.5e-115 |
| ACB46195.1 | polyketide\_synthase | BGC0000989 | Epothilone\_biosynthetic\_gene\_cluster | 29 | 54.2342342342 | 414 | 7.2e-115 |
| ABX60152.1 | polyketide\_synthase | BGC0000978 | Cylindrospermopsin\_biosynthetic\_gene\_cluster | 30 | 53.963963964 | 413 | 1.2e-114 |
| ABB86410.1 | GelC | BGC0000067 | Geldanamycin\_biosynthetic\_gene\_cluster | 29 | 55.5855855856 | 413 | 1.2e-114 |
| AGI99497.1 | type\_I\_polyketide\_synthase | BGC0001004 | Lobophorin\_biosynthetic\_gene\_cluster | 33 | 43.4684684685 | 413 | 2.1e-114 |
| BAQ25507.1 | type\_I\_polyketide\_synthase | BGC0001288 | Maklamicin\_biosynthetic\_gene\_cluster | 32 | 46.1711711712 | 413 | 2.1e-114 |
| CAQ34920.1 | polyketide\_synthase | BGC0000986 | Dkxanthene\_biosynthetic\_gene\_cluster | 30 | 53.963963964 | 412 | 2.7e-114 |
| ACR33077.1 | polyketide\_synthase | BGC0000017 | Anatoxin\_biosynthetic\_gene\_cluster | 27 | 54.4594594595 | 411 | 4.7e-114 |
| CAQ34919.1 | polyketide\_synthase | BGC0000986 | Dkxanthene\_biosynthetic\_gene\_cluster | 32 | 47.4774774775 | 411 | 4.7e-114 |
| BAJ16468.1 | polyketide\_synthase | BGC0000058 | FD-891\_biosynthetic\_gene\_cluster | 31 | 46.3963963964 | 411 | 6.1e-114 |
| ABK32287.1 | JerA | BGC0000080 | Jerangolid\_biosynthetic\_gene\_cluster | 32 | 42.972972973 | 411 | 8e-114 |
| AAF62884.1 | EpoE | BGC0000991 | Epothilone\_biosynthetic\_gene\_cluster | 33 | 42.6576576577 | 410 | 1e-113 |
| ADB12492.1 | EpoE | BGC0000990 | Epothilone\_biosynthetic\_gene\_cluster | 33 | 42.6576576577 | 410 | 1e-113 |
| AAF26922.1 | polyketide\_synthase | BGC0000988 | Epothilone\_biosynthetic\_gene\_cluster | 33 | 42.6576576577 | 410 | 1.4e-113 |
| ACB46196.1 | polyketide\_synthase | BGC0000989 | Epothilone\_biosynthetic\_gene\_cluster | 33 | 42.6576576577 | 409 | 2.3e-113 |
| AFV96138.1 | polyketide\_synthase | BGC0001064 | Cylindrocyclophane\_biosynthetic\_gene\_cluster | 32 | 43.3783783784 | 409 | 2.3e-113 |
| ABK32255.1 | AmbA | BGC0000014 | Ambruticin\_biosynthetic\_gene\_cluster | 32 | 42.972972973 | 408 | 4e-113 |
| ABM21570.1 | CrpB | BGC0000975 | Cryptophycin\_biosynthetic\_gene\_cluster | 32 | 42.9279279279 | 408 | 5.2e-113 |
| AAU04878.1 | polyketide\_synthase | BGC0000365 | Glycopeptidolipid\_biosynthetic\_gene\_cluster | 28 | 54.0540540541 | 408 | 5.2e-113 |
| ABK32257.1 | AmbC | BGC0000014 | Ambruticin\_biosynthetic\_gene\_cluster | 30 | 46.1711711712 | 408 | 6.7e-113 |
| AGC09484.1 | LobS1 | BGC0001183 | Lobophorin\_biosynthetic\_gene\_cluster | 32 | 43.4684684685 | 408 | 6.7e-113 |
| CBD77734.1 | polyketide\_synthase | BGC0000974 | Crocacin\_biosynthetic\_gene\_cluster | 32 | 42.2072072072 | 407 | 1.2e-112 |
| ABJ97439.1 | MerC | BGC0001012 | Meridamycin\_biosynthetic\_gene\_cluster | 30 | 47.0720720721 | 406 | 1.5e-112 |
| BAJ16467.1 | polyketide\_synthase | BGC0000058 | FD-891\_biosynthetic\_gene\_cluster | 30 | 46.6666666667 | 406 | 2e-112 |
| ctg1\_orf20 |  | BGC0001013 | Meridamycin\_biosynthetic\_gene\_cluster | 29 | 53.6486486486 | 406 | 2.6e-112 |
| ABC87509.1 | polyketide\_synthase | BGC0001011 | Meridamycin\_biosynthetic\_gene\_cluster | 29 | 53.6486486486 | 406 | 2.6e-112 |
| BAO66529.1 | type\_I\_polyketide\_synthase | BGC0000042 | Cremimycin\_biosynthetic\_gene\_cluster | 33 | 43.0630630631 | 405 | 3.3e-112 |
| AAC69329.1 | type\_I\_polyketide\_synthase\_PikAI | BGC0000094 | Methymycin\_/\_pikromycin\_biosynthetic\_gene\_cluster | 27 | 61.4864864865 | 405 | 3.3e-112 |
| ADH04639.1 | TgaA | BGC0001051 | Thuggacin\_biosynthetic\_gene\_cluster | 31 | 46.0810810811 | 405 | 4.4e-112 |
| BAF02924.1 | type\_I\_polyketide\_synthase | BGC0000073 | Halstoctacosanolide\_biosynthetic\_gene\_cluster | 31 | 45.7207207207 | 404 | 7.5e-112 |
| AAO06918.1 | GdmAIII | BGC0000066 | Geldanamycin\_biosynthetic\_gene\_cluster | 29 | 55.4954954955 | 404 | 9.7e-112 |
| AAT70096.1 | CurA | BGC0001165 | Curacin\_biosynthetic\_gene\_cluster | 31 | 43.1081081081 | 404 | 9.7e-112 |
| AEE88289.1 | CurA | BGC0000976 | Curacin\_biosynthetic\_gene\_cluster | 31 | 43.1081081081 | 404 | 9.7e-112 |
| BAK64649.1 | polyketide\_synthase | BGC0000135 | Reveromycin\_biosynthetic\_gene\_cluster | 30 | 46.0810810811 | 404 | 9.7e-112 |
| ABK32289.1 | JerC | BGC0000080 | Jerangolid\_biosynthetic\_gene\_cluster | 31 | 46.2162162162 | 402 | 2.8e-111 |
| ctg1\_orf523 |  | BGC0001199 | Akaeolide\_biosynthetic\_gene\_cluster | 30 | 46.3513513514 | 402 | 2.8e-111 |
| ACR33079.1 | polyketide\_synthase | BGC0000017 | Anatoxin\_biosynthetic\_gene\_cluster | 31 | 42.8378378378 | 401 | 4.8e-111 |
| CAD89773.1 | MelB\_protein | BGC0001009 | Melithiazol\_biosynthetic\_gene\_cluster | 32 | 43.1081081081 | 401 | 4.8e-111 |
| ABK32256.1 | AmbB | BGC0000014 | Ambruticin\_biosynthetic\_gene\_cluster | 31 | 42.8378378378 | 401 | 6.3e-111 |
| OAQ83765.1 | KR\_domain-containing\_protein | BGC0001358 | Leucinostatins\_biosynthetic\_gene\_cluster | 30 | 43.5585585586 | 401 | 6.3e-111 |
| AAC69330.1 | type\_I\_polyketide\_synthase\_PikAII | BGC0000094 | Methymycin\_/\_pikromycin\_biosynthetic\_gene\_cluster | 29 | 46.1711711712 | 401 | 8.2e-111 |
| ACB46471.1 | polyketide\_synthase | BGC0000082 | Kijanimicin\_biosynthetic\_gene\_cluster | 28 | 58.963963964 | 401 | 8.2e-111 |
| ABB86408.1 | GelA | BGC0000067 | Geldanamycin\_biosynthetic\_gene\_cluster | 31 | 46.1261261261 | 400 | 1.1e-110 |
| AEP40934.1 | polyketide\_synthase\_type\_I | BGC0000021 | Apoptolidin\_biosynthetic\_gene\_cluster | 30 | 46.1711711712 | 400 | 1.1e-110 |
| AAT70108.1 | CurM | BGC0001165 | Curacin\_biosynthetic\_gene\_cluster | 31 | 43.1981981982 | 400 | 1.4e-110 |
| ACV42478.1 | polyketide\_synthase | BGC0000043 | Curacin\_A\_biosynthetic\_gene\_cluster | 31 | 43.1981981982 | 400 | 1.4e-110 |
| AEE88277.1 | CurM | BGC0000976 | Curacin\_biosynthetic\_gene\_cluster | 31 | 43.1981981982 | 400 | 1.4e-110 |
| ACB46487.1 | polyketide\_synthase | BGC0000082 | Kijanimicin\_biosynthetic\_gene\_cluster | 32 | 45.990990991 | 399 | 1.8e-110 |
| AEP40936.1 | polyketide\_synthase\_type\_I | BGC0000021 | Apoptolidin\_biosynthetic\_gene\_cluster | 27 | 55.0 | 398 | 4.1e-110 |
| AMK48247.1 | 6-methylsalicylic\_acid\_synthase | BGC0001351\_c2 | Cahuitamycins\_biosynthetic\_gene\_cluster | 32 | 46.3513513514 | 398 | 5.3e-110 |
| ADB12493.1 | EpoF | BGC0000990 | Epothilone\_biosynthetic\_gene\_cluster | 28 | 57.027027027 | 397 | 9.1e-110 |
| BAE93730.1 | type\_I\_polyketide\_synthase | BGC0000164 | Tetronomycin\_biosynthetic\_gene\_cluster | 28 | 55.2252252252 | 397 | 9.1e-110 |
| AFV30250.1 | polyketide\_synthase | BGC0000075 | Hygrocin\_biosynthetic\_gene\_cluster | 30 | 46.3513513514 | 396 | 1.6e-109 |
| ACN64831.1 | PokM1 | BGC0001061 | Polyketomycin\_biosynthetic\_gene\_cluster | 32 | 46.5315315315 | 396 | 1.6e-109 |
| AAX98184.1 | polyketide\_synthase\_type\_I | BGC0000052 | ECO-02301\_biosynthetic\_gene\_cluster | 27 | 59.1441441441 | 396 | 1.6e-109 |
| ABJ97437.1 | MerA | BGC0001012 | Meridamycin\_biosynthetic\_gene\_cluster | 30 | 44.5945945946 | 396 | 2e-109 |
| ABK32288.1 | JerB | BGC0000080 | Jerangolid\_biosynthetic\_gene\_cluster | 31 | 42.8378378378 | 395 | 3.5e-109 |
| ABB05105.1 | LipPks4 | BGC0001003 | Lipomycin\_biosynthetic\_gene\_cluster | 31 | 46.3513513514 | 395 | 3.5e-109 |
| CAC20931.1 | PimS1\_protein | BGC0000125 | Pimaricin\_biosynthetic\_gene\_cluster | 30 | 46.1711711712 | 395 | 3.5e-109 |
| AEE88284.1 | CurF | BGC0000976 | Curacin\_biosynthetic\_gene\_cluster | 28 | 55.3153153153 | 394 | 5.9e-109 |
| WP\_043476516.1 | beta-ketoacyl\_synthase | BGC0001232 | Leucanicidin\_biosynthetic\_gene\_cluster | 31 | 46.1711711712 | 394 | 5.9e-109 |
| AAX98188.1 | polyketide\_synthase\_type\_I | BGC0000052 | ECO-02301\_biosynthetic\_gene\_cluster | 31 | 46.1261261261 | 394 | 7.7e-109 |
| AEU11006.1 | NpnB | BGC0001029 | Nostophycin\_biosynthetic\_gene\_cluster | 31 | 42.972972973 | 394 | 1e-108 |
| AAW03325.1 | CtaB | BGC0000982 | Cystothiazole\_A\_biosynthetic\_gene\_cluster | 31 | 42.4774774775 | 393 | 1.3e-108 |
| ctg1\_orf21 |  | BGC0001013 | Meridamycin\_biosynthetic\_gene\_cluster | 28 | 54.0540540541 | 393 | 1.7e-108 |
| ABC87510.1 | polyketide\_synthase | BGC0001011 | Meridamycin\_biosynthetic\_gene\_cluster | 28 | 54.0540540541 | 393 | 1.7e-108 |
| AAT70101.1 | CurF | BGC0001165 | Curacin\_biosynthetic\_gene\_cluster | 28 | 55.3153153153 | 393 | 2.2e-108 |
| AAF62885.1 | EpoF | BGC0000991 | Epothilone\_biosynthetic\_gene\_cluster | 28 | 56.981981982 | 393 | 2.2e-108 |
| CAD89777.1 | MelF\_protein | BGC0001009 | Melithiazol\_biosynthetic\_gene\_cluster | 31 | 42.2972972973 | 392 | 2.9e-108 |
| BAF85844.1 | modular\_polyketide\_synthase | BGC0000109 | Nemadectin\_biosynthetic\_gene\_cluster | 29 | 56.5315315315 | 392 | 2.9e-108 |
| AHH34186.1 | polyketide\_synthase | BGC0001161 | 1-heptadecene\_biosynthetic\_gene\_cluster | 32 | 43.6036036036 | 392 | 3.8e-108 |
| AAZ94390.1 | modular\_polyketide\_synthase | BGC0000040 | Concanamycin\_A\_biosynthetic\_gene\_cluster | 31 | 45.3603603604 | 391 | 5e-108 |
| AFL48529.1 | laidlomycin\_polyketide\_synthase\_(module\_5\_and\_module\_6) | BGC0000084 | Laidlomycin\_biosynthetic\_gene\_cluster | 31 | 45.8108108108 | 391 | 8.5e-108 |
| AAK73514.1 | AmphC | BGC0000015 | Amphotericin\_biosynthetic\_gene\_cluster | 29 | 46.4414414414 | 391 | 8.5e-108 |
| AEU11005.1 | NpnA | BGC0001029 | Nostophycin\_biosynthetic\_gene\_cluster | 30 | 42.6576576577 | 390 | 1.5e-107 |
| AAQ84145.1 | Plm5 | BGC0000123 | Phoslactomycin\_B\_biosynthetic\_gene\_cluster | 29 | 46.1261261261 | 390 | 1.5e-107 |
| ABJ97438.1 | MerB | BGC0001012 | Meridamycin\_biosynthetic\_gene\_cluster | 29 | 46.3513513514 | 389 | 1.9e-107 |
| AAQ82566.1 | FscF | BGC0000034 | Candicidin\_biosynthetic\_gene\_cluster | 29 | 49.1441441441 | 389 | 1.9e-107 |
| ACO94483.1 | polyketide\_synthase\_type\_I | BGC0000097 | ML-449\_biosynthetic\_gene\_cluster | 27 | 54.4144144144 | 389 | 2.5e-107 |
| CAA16183.1 | polyketide\_synthase | BGC0001063 | Undecylprodigiosin\_biosynthetic\_gene\_cluster | 32 | 42.7927927928 | 389 | 3.2e-107 |
| ACB37740.1 | putative\_type\_I\_polyketide\_synthase | BGC0000162 | Tetrocarcin\_A\_biosynthetic\_gene\_cluster | 30 | 45.4954954955 | 389 | 3.2e-107 |
| AAX98190.1 | polyketide\_synthase\_type\_I | BGC0000052 | ECO-02301\_biosynthetic\_gene\_cluster | 30 | 43.2432432432 | 389 | 3.2e-107 |
| BAE93731.1 | type\_I\_polyketide\_synthase | BGC0000164 | Tetronomycin\_biosynthetic\_gene\_cluster | 27 | 55.0900900901 | 388 | 4.2e-107 |
| ADN13832.1 | Beta-ketoacyl\_synthase | BGC0001164 | 1-heptadecene\_biosynthetic\_gene\_cluster | 32 | 43.018018018 | 388 | 4.2e-107 |
| AAS98781.1 | JamJ | BGC0001001 | Jamaicamide\_biosynthetic\_gene\_cluster | 28 | 55.1801801802 | 388 | 5.5e-107 |
| ADX66472.1 | ScnS1 | BGC0000108 | Natamycin\_biosynthetic\_gene\_cluster | 30 | 45.6756756757 | 388 | 5.5e-107 |
| AAW03329.1 | CtaF | BGC0000982 | Cystothiazole\_A\_biosynthetic\_gene\_cluster | 31 | 42.7027027027 | 388 | 5.5e-107 |
| AKD43522.1 | Type\_I\_polyketide\_synthase | BGC0001409 | Dutomycin\_biosynthetic\_gene\_cluster | 31 | 46.7117117117 | 388 | 5.5e-107 |
| AEP40940.1 | polyketide\_synthase\_type\_I | BGC0000021 | Apoptolidin\_biosynthetic\_gene\_cluster | 30 | 42.4774774775 | 388 | 7.2e-107 |
| AEZ53946.1 | polyketide\_synthase | BGC0000144 | Salinomycin\_biosynthetic\_gene\_cluster | 30 | 45.5855855856 | 388 | 7.2e-107 |
| CAE02602.1 | polyketide\_synthase\_type\_I | BGC0000024 | Aureothin\_biosynthetic\_gene\_cluster | 28 | 54.7747747748 | 387 | 9.4e-107 |
| BAP34763.1 | type\_I\_polyketide\_synthase | BGC0000078 | Incednine\_biosynthetic\_gene\_cluster | 29 | 49.009009009 | 387 | 9.4e-107 |
| ADH04640.1 | TgaB | BGC0001051 | Thuggacin\_biosynthetic\_gene\_cluster | 32 | 42.8828828829 | 387 | 1.2e-106 |
| ADC79618.1 | BafAIII | BGC0000028 | Bafilomycin\_biosynthetic\_gene\_cluster | 30 | 45.9459459459 | 386 | 1.6e-106 |
| BAK64638.1 | polyketide\_synthase | BGC0000135 | Reveromycin\_biosynthetic\_gene\_cluster | 30 | 46.8018018018 | 386 | 1.6e-106 |
| CAC22145.1 | putative\_type\_I\_polyketide\_synthase | BGC0000038 | Coelimycin\_biosynthetic\_gene\_cluster | 29 | 48.3333333333 | 385 | 3.6e-106 |
| CUW01174.1 | Type\_I\_polyketide\_synthase | BGC0001359 | PM100117\_/\_PM100118\_biosynthetic\_gene\_cluster | 28 | 54.6396396396 | 385 | 4.7e-106 |
| ALA09371.1 | type\_I\_modular\_PKS | BGC0001303 | Lobosamide\_biosynthetic\_gene\_cluster | 30 | 45.9459459459 | 385 | 4.7e-106 |
| AAG23265.1 | polyketide\_synthase\_extender\_module\_2 | BGC0000148 | Spinosad\_biosynthetic\_gene\_cluster | 28 | 54.2792792793 | 384 | 6.1e-106 |
| BAJ16470.1 | polyketide\_synthase | BGC0000058 | FD-891\_biosynthetic\_gene\_cluster | 30 | 43.963963964 | 384 | 8e-106 |
| ABI94380.1 | tautomycetin\_biosynthetic\_PKS | BGC0000157 | Tautomycetin\_biosynthetic\_gene\_cluster | 28 | 55.2252252252 | 384 | 8e-106 |
| AAO62582.1 | polyketide\_synthase\_peptide\_sythetase\_fusion\_protein | BGC0001016 | Microcystin\_biosynthetic\_gene\_cluster | 31 | 42.7927927928 | 384 | 1e-105 |
| BAO66542.1 | type\_I\_polyketide\_synthase | BGC0000042 | Cremimycin\_biosynthetic\_gene\_cluster | 30 | 45.5405405405 | 384 | 1e-105 |
| BAH02268.1 | polyketide\_synthase | BGC0000126 | Pladienolide\_biosynthetic\_gene\_cluster | 30 | 46.3063063063 | 383 | 2.3e-105 |
| ctg1\_orf11 |  | BGC0000053 | Elaiophylin\_biosynthetic\_gene\_cluster | 29 | 46.3513513514 | 383 | 2.3e-105 |
| CAJ88177.1 | putative\_type\_I\_polyketide\_synthase | BGC0000151 | Stambomycin\_biosynthetic\_gene\_cluster | 30 | 45.990990991 | 382 | 3e-105 |
| AHN85651.1 | Phn2 | BGC0000122 | Phenylnannolone\_A\_biosynthetic\_gene\_cluster | 30 | 46.3513513514 | 382 | 3e-105 |
| AAK19883.1 | soraphen\_polyketide\_synthase\_A | BGC0000147 | Soraphen\_biosynthetic\_gene\_cluster | 27 | 54.6396396396 | 382 | 3e-105 |
| ABV91287.1 | type\_I\_modular\_polyketide\_synthase | BGC0000158 | Tautomycetin\_biosynthetic\_gene\_cluster | 28 | 55.2252252252 | 382 | 3e-105 |
| CUW01175.1 | Type\_I\_polyketide\_synthase | BGC0001359 | PM100117\_/\_PM100118\_biosynthetic\_gene\_cluster | 27 | 55.045045045 | 382 | 3e-105 |
| ACB37755.1 | putative\_type\_I\_polyketide\_synthase | BGC0000162 | Tetrocarcin\_A\_biosynthetic\_gene\_cluster | 28 | 46.0810810811 | 382 | 4e-105 |
| AJW65409.1 | type\_I\_modular\_polyketide\_synthase | BGC0001195 | Nocardiopsin\_biosynthetic\_gene\_cluster | 29 | 46.036036036 | 382 | 4e-105 |
| BAF02922.1 | type\_I\_polyketide\_synthase | BGC0000073 | Halstoctacosanolide\_biosynthetic\_gene\_cluster | 29 | 46.0810810811 | 382 | 4e-105 |
| BAD08359.1 | polyketide\_synthase\_modules\_5-6 | BGC0000167 | Vicenistatin\_biosynthetic\_gene\_cluster | 27 | 54.5045045045 | 381 | 5.2e-105 |
| ADX66461.1 | ScnS2 | BGC0000108 | Natamycin\_biosynthetic\_gene\_cluster | 31 | 42.972972973 | 381 | 6.8e-105 |
| ABX60163.1 | polyketide\_synthase | BGC0000978 | Cylindrospermopsin\_biosynthetic\_gene\_cluster | 30 | 43.7387387387 | 381 | 6.8e-105 |
| BAF85839.1 | modular\_polyketide\_synthase | BGC0000109 | Nemadectin\_biosynthetic\_gene\_cluster | 30 | 45.8558558559 | 381 | 6.8e-105 |
| AFU82617.1 | polyketide\_synthase | BGC0000998 | Guadinomine\_biosynthetic\_gene\_cluster | 31 | 42.2072072072 | 381 | 8.8e-105 |
| BAR73007.1 | putative\_PKS\_(ACP-KS-AT-DH-KR-ACP-KS-AT-DH-ER-KR-ACP) | BGC0001194 | Hitachimycin\_biosynthetic\_gene\_cluster | 28 | 54.0990990991 | 381 | 8.8e-105 |
| CUW01177.1 | Type\_I\_polyketide\_synthase | BGC0001359\_c2 | PM100117\_/\_PM100118\_biosynthetic\_gene\_cluster | 27 | 57.4324324324 | 380 | 1.2e-104 |
| BAB69196.1 | modular\_polyketide\_synthase | BGC0000117 | Oligomycin\_biosynthetic\_gene\_cluster | 30 | 42.5225225225 | 380 | 1.2e-104 |
| BAD08373.1 | polyketide\_synthase\_modules\_1-3 | BGC0000167 | Vicenistatin\_biosynthetic\_gene\_cluster | 28 | 50.990990991 | 380 | 1.2e-104 |
| AHH99925.1 | PKS\_I | BGC0000002 | Aculeximycin\_biosynthetic\_gene\_cluster | 30 | 45.4954954955 | 380 | 1.5e-104 |
| AFL48527.1 | laidlomycin\_polyketide\_synthase\_(module\_3\_and\_module\_4) | BGC0000084 | Laidlomycin\_biosynthetic\_gene\_cluster | 29 | 56.1261261261 | 379 | 2e-104 |
| ADC45586.1 | modular\_polyketide\_synthase | BGC0000093 | Meilingmycin\_biosynthetic\_gene\_cluster | 31 | 44.954954955 | 379 | 2e-104 |
| BAF85843.1 | modular\_polyketide\_synthase | BGC0000109 | Nemadectin\_biosynthetic\_gene\_cluster | 30 | 46.2612612613 | 379 | 2e-104 |
| ACO94456.1 | polyketide\_synthase\_type\_I | BGC0000029 | BE-14106\_biosynthetic\_gene\_cluster | 27 | 54.5045045045 | 379 | 2.6e-104 |
| CAQ18832.1 | polyketide\_synthase | BGC0000954 | Ajudazol\_biosynthetic\_gene\_cluster | 28 | 55.4954954955 | 379 | 2.6e-104 |
| ADF88277.1 | polyketide\_synthase | BGC0000981 | Cylindrospermopsin\_biosynthetic\_gene\_cluster | 30 | 43.7387387387 | 379 | 2.6e-104 |
| AHB82053.1 | polyketide\_synthase | BGC0001019 | Microsclerodermins\_biosynthetic\_gene\_cluster | 30 | 42.2072072072 | 378 | 4.4e-104 |
| ALP32045.1 | CycE | BGC0001293 | Cyclizidine\_biosynthetic\_gene\_cluster | 29 | 47.1171171171 | 378 | 4.4e-104 |
| CUW01172.1 | Type\_I\_polyketide\_synthase | BGC0001359 | PM100117\_/\_PM100118\_biosynthetic\_gene\_cluster | 28 | 45.990990991 | 378 | 5.7e-104 |
| ACB46197.1 | polyketide\_synthase | BGC0000989 | Epothilone\_biosynthetic\_gene\_cluster | 28 | 57.027027027 | 378 | 5.7e-104 |
| AAQ82567.1 | FscE | BGC0000061 | FR-008\_biosynthetic\_gene\_cluster | 30 | 45.990990991 | 377 | 9.8e-104 |
| OAQ83760.1 | polyketide\_synthase | BGC0001358 | Leucinostatins\_biosynthetic\_gene\_cluster | 30 | 39.6396396396 | 377 | 9.8e-104 |
| AAG23263.1 | polyketide\_synthase\_extender\_modules\_5-7 | BGC0000148 | Spinosad\_biosynthetic\_gene\_cluster | 27 | 54.8648648649 | 377 | 1.3e-103 |
| AAY42396.1 | HctD | BGC0001000 | Hectochlorin\_biosynthetic\_gene\_cluster | 31 | 43.018018018 | 377 | 1.3e-103 |
| BAF02921.1 | type\_I\_polyketide\_synthase | BGC0000073 | Halstoctacosanolide\_biosynthetic\_gene\_cluster | 30 | 43.0630630631 | 376 | 1.7e-103 |
| ACN69991.1 | polyketide\_synthase | BGC0000079 | Indanomycin\_biosynthetic\_gene\_cluster | 27 | 54.3693693694 | 376 | 1.7e-103 |
| CAC20921.1 | PimS2\_protein | BGC0000125 | Pimaricin\_biosynthetic\_gene\_cluster | 31 | 42.972972973 | 376 | 1.7e-103 |
| CUW01173.1 | Type\_I\_polyketide\_synthase | BGC0001359 | PM100117\_/\_PM100118\_biosynthetic\_gene\_cluster | 29 | 46.0810810811 | 376 | 2.2e-103 |
| ACR50773.1 | polyketide\_synthase | BGC0000163 | Tetronasin\_biosynthetic\_gene\_cluster | 28 | 46.2612612613 | 376 | 2.2e-103 |
| ABB05104.1 | LipPks3 | BGC0001003 | Lipomycin\_biosynthetic\_gene\_cluster | 30 | 46.4864864865 | 376 | 2.2e-103 |
| AEH42491.1 | polyketide\_synthase | BGC0000032 | Calcimycin\_biosynthetic\_gene\_cluster | 29 | 42.7927927928 | 376 | 2.2e-103 |
| CAJ88176.1 | putative\_polyketide\_synthase\_B | BGC0000151 | Stambomycin\_biosynthetic\_gene\_cluster | 30 | 45.990990991 | 376 | 2.8e-103 |
| AEU17899.1 | putative\_type\_I\_PKS | BGC0001072 | Simocyclinone\_biosynthetic\_gene\_cluster | 31 | 42.8378378378 | 376 | 2.8e-103 |
| AAF26923.1 | polyketide\_synthase | BGC0000988 | Epothilone\_biosynthetic\_gene\_cluster | 29 | 46.5315315315 | 376 | 2.8e-103 |
| CAD29793.1 | polyketide\_synthase\_type\_I | BGC0001015 | Microcystin\_biosynthetic\_gene\_cluster | 29 | 45.990990991 | 375 | 3.7e-103 |
| BAO66528.1 | type\_I\_polyketide\_synthase | BGC0000042 | Cremimycin\_biosynthetic\_gene\_cluster | 29 | 46.0810810811 | 375 | 4.8e-103 |
| BAB69195.1 | modular\_polyketide\_synthase | BGC0000117 | Oligomycin\_biosynthetic\_gene\_cluster | 30 | 43.4234234234 | 375 | 4.8e-103 |
| AAS98777.1 | JamE | BGC0001001 | Jamaicamide\_biosynthetic\_gene\_cluster | 31 | 43.3333333333 | 374 | 6.3e-103 |
| ABI94379.1 | tautomycetin\_biosynthetic\_PKS | BGC0000157 | Tautomycetin\_biosynthetic\_gene\_cluster | 28 | 50.4504504505 | 374 | 6.3e-103 |
| BAC76492.1 | lankamycin\_synthase\_LkmAII | BGC0000085 | Lankamycin\_biosynthetic\_gene\_cluster | 30 | 46.1711711712 | 374 | 8.3e-103 |
| CAJ88187.2 | putative\_type\_I\_modular\_polyketide\_synthase | BGC0000151 | Stambomycin\_biosynthetic\_gene\_cluster | 26 | 54.9099099099 | 374 | 8.3e-103 |
| AEZ64505.1 | Herb | BGC0001065 | Herboxidiene\_biosynthetic\_gene\_cluster | 30 | 46.3513513514 | 374 | 8.3e-103 |
| AAM70355.1 | CalO5 | BGC0000033 | Calicheamicin\_biosynthetic\_gene\_cluster | 30 | 46.036036036 | 373 | 1.4e-102 |
| ACR50785.1 | polyketide\_synthase | BGC0000163 | Tetronasin\_biosynthetic\_gene\_cluster | 31 | 45.4504504505 | 373 | 1.4e-102 |
| AEZ54379.1 | PieA6 | BGC0000124 | Piericidin\_A1\_biosynthetic\_gene\_cluster | 30 | 43.1981981982 | 373 | 1.4e-102 |
| BAP34734.1 | type\_I\_polyketide\_synthase | BGC0000078 | Incednine\_biosynthetic\_gene\_cluster | 29 | 45.8558558559 | 373 | 1.8e-102 |
| AGC09499.1 | LobS4 | BGC0001183 | Lobophorin\_biosynthetic\_gene\_cluster | 29 | 46.7567567568 | 373 | 1.8e-102 |
| CAC20919.1 | PimS4\_protein | BGC0000125 | Pimaricin\_biosynthetic\_gene\_cluster | 29 | 47.8828828829 | 373 | 1.8e-102 |
| BAB69194.1 | modular\_polyketide\_synthase | BGC0000117 | Oligomycin\_biosynthetic\_gene\_cluster | 29 | 45.9009009009 | 373 | 2.4e-102 |
| AAK73502.1 | AmphJ | BGC0000015 | Amphotericin\_biosynthetic\_gene\_cluster | 26 | 59.1441441441 | 373 | 2.4e-102 |
| ABV91286.1 | type\_I\_modular\_polyketide\_synthase | BGC0000158 | Tautomycetin\_biosynthetic\_gene\_cluster | 28 | 50.4504504505 | 373 | 2.4e-102 |
| ACF35445.1 | MbcAI | BGC0000090 | Macbecin\_biosynthetic\_gene\_cluster | 30 | 46.0810810811 | 372 | 3.1e-102 |
| BAC68125.1 | modular\_polyketide\_synthase | BGC0000059 | Filipin\_biosynthetic\_gene\_cluster | 29 | 42.7477477477 | 372 | 3.1e-102 |
| CAO85897.1 | modular\_polyketide\_synthase\_NorB | BGC0000110 | Neoaureothin\_biosynthetic\_gene\_cluster | 27 | 54.7747747748 | 372 | 4.1e-102 |
| AAQ90173.1 | polyketide\_synthase\_type\_I | BGC0000128 | Pyoluteorin\_biosynthetic\_gene\_cluster | 30 | 42.2972972973 | 372 | 4.1e-102 |
| AAF71766.1 | NysI | BGC0000115 | Nystatin\_biosynthetic\_gene\_cluster | 30 | 46.1711711712 | 371 | 9.1e-102 |
| AHH34189.1 | polyketide\_synthase | BGC0001162 | 1-heptadecene\_biosynthetic\_gene\_cluster | 31 | 44.5945945946 | 371 | 9.1e-102 |
| CAQ52622.1 | type\_I\_polyketide\_synthase,\_modules\_4-5 | BGC0001066 | Kendomycin\_biosynthetic\_gene\_cluster | 29 | 48.4684684685 | 371 | 9.1e-102 |
| AEZ64504.1 | Herc | BGC0001065 | Herboxidiene\_biosynthetic\_gene\_cluster | 29 | 46.5315315315 | 371 | 9.1e-102 |
| AAQ82568.1 | FscD | BGC0000034 | Candicidin\_biosynthetic\_gene\_cluster | 30 | 43.1981981982 | 370 | 1.2e-101 |
| ACN69990.1 | polyketide\_synthase | BGC0000079 | Indanomycin\_biosynthetic\_gene\_cluster | 29 | 46.1711711712 | 370 | 1.6e-101 |
| ACR50775.1 | polyketide\_synthase | BGC0000163 | Tetronasin\_biosynthetic\_gene\_cluster | 28 | 45.8108108108 | 369 | 2e-101 |
| ACY06287.1 | type\_I\_polyketide\_synthase | BGC0001042 | Sanglifehrin\_A\_biosynthetic\_gene\_cluster | 30 | 42.6126126126 | 369 | 2e-101 |
| CAL58685.1 | polyketide\_synthase | BGC0000149 | Spirangien\_biosynthetic\_gene\_cluster | 28 | 47.8378378378 | 369 | 2e-101 |
| AFV30248.1 | polyketide\_synthase | BGC0000075 | Hygrocin\_biosynthetic\_gene\_cluster | 29 | 47.1621621622 | 369 | 2e-101 |
| AAG13919.1 | megalomicin\_6-deoxyerythronolide\_B\_synthase\_3 | BGC0000092 | Megalomicin\_biosynthetic\_gene\_cluster | 31 | 42.6576576577 | 369 | 2.7e-101 |
| AAZ94387.1 | modular\_polyketide\_synthase | BGC0000040 | Concanamycin\_A\_biosynthetic\_gene\_cluster | 31 | 43.018018018 | 369 | 2.7e-101 |
| AAX98191.1 | polyketide\_synthase\_type\_I | BGC0000052 | ECO-02301\_biosynthetic\_gene\_cluster | 28 | 46.4414414414 | 369 | 3.5e-101 |
| ADF88275.1 | polyketide\_synthase | BGC0000981 | Cylindrospermopsin\_biosynthetic\_gene\_cluster | 30 | 42.7927927928 | 369 | 3.5e-101 |
| AEZ64503.1 | Herd | BGC0001065 | Herboxidiene\_biosynthetic\_gene\_cluster | 30 | 45.990990991 | 369 | 3.5e-101 |
| ABO15860.1 | polyketide\_synthase | BGC0000130 | Pyrrolomycin\_biosynthetic\_gene\_cluster | 31 | 41.9369369369 | 368 | 4.5e-101 |
| AJW65407.1 | type\_I\_modular\_polyketide\_synthase | BGC0001195 | Nocardiopsin\_biosynthetic\_gene\_cluster | 29 | 46.0810810811 | 368 | 4.5e-101 |
| ADB23403.1 | polyketide\_synthase\_type\_I | BGC0001062 | TLN-05220\_biosynthetic\_gene\_cluster | 28 | 54.3693693694 | 368 | 4.5e-101 |
| AAY28227.1 | HbmAIII | BGC0000074 | Herbimycin\_biosynthetic\_gene\_cluster | 29 | 46.4414414414 | 368 | 4.5e-101 |
| EFY94490.1 | polyketide\_synthase,\_putative | BGC0000337 | Destruxins\_biosynthetic\_gene\_cluster | 31 | 39.7747747748 | 368 | 4.5e-101 |
| CAL58686.1 | polyketide\_synthase | BGC0000149 | Spirangien\_biosynthetic\_gene\_cluster | 28 | 48.5585585586 | 368 | 7.7e-101 |
| AAZ77693.1 | ChlA1 | BGC0000036 | Chlorothricin\_biosynthetic\_gene\_cluster | 29 | 46.3963963964 | 368 | 7.7e-101 |
| ABP55210.1 | beta-ketoacyl\_synthase | BGC0000142 | Salinilactam\_biosynthetic\_gene\_cluster | 30 | 46.1261261261 | 367 | 1e-100 |
| AAX98189.1 | polyketide\_synthase\_type\_I | BGC0000052 | ECO-02301\_biosynthetic\_gene\_cluster | 28 | 49.7747747748 | 367 | 1e-100 |
| CCE88377.1 | non-ribosomal\_peptide\_synthetase/polyketide\_synthase | BGC0001034 | Pellasoren\_biosynthetic\_gene\_cluster | 31 | 41.2612612613 | 367 | 1.3e-100 |
| BAD08358.1 | polyketide\_synthase\_modules\_4 | BGC0000167 | Vicenistatin\_biosynthetic\_gene\_cluster | 29 | 46.3513513514 | 366 | 1.7e-100 |
| BAD08360.1 | polyketide\_synthase\_modules\_7-8 | BGC0000167 | Vicenistatin\_biosynthetic\_gene\_cluster | 29 | 46.2612612613 | 366 | 1.7e-100 |
| AFV30251.1 | polyketide\_synthase | BGC0000075 | Hygrocin\_biosynthetic\_gene\_cluster | 31 | 42.6576576577 | 366 | 2.3e-100 |
| EHA22196.1 | polyketide\_synthase | BGC0000170 | Yanuthone\_D\_biosynthetic\_gene\_cluster | 29 | 46.4864864865 | 366 | 2.3e-100 |
| AAK83194.1 | polyketide\_synthase | BGC0000026 | Avilamycin\_A\_biosynthetic\_gene\_cluster | 29 | 48.6036036036 | 366 | 2.3e-100 |
| ABV83229.1 | CppB | BGC0000116 | Nystatin-like\_Pseudonocardia\_polyene\_biosynthetic\_gene\_cluster | 32 | 42.2972972973 | 366 | 2.3e-100 |
| AAG23262.1 | polyketide\_synthase\_extender\_modules\_8-10 | BGC0000148 | Spinosad\_biosynthetic\_gene\_cluster | 29 | 46.5765765766 | 366 | 2.3e-100 |
| AHA12078.1 | polyketide\_synthase\_type\_1 | BGC0001172 | Chlorizidine\_A\_biosynthetic\_gene\_cluster | 31 | 43.018018018 | 366 | 2.9e-100 |
| ALA09356.1 | type\_I\_modular\_PKS | BGC0001303 | Lobosamide\_biosynthetic\_gene\_cluster | 31 | 42.1621621622 | 366 | 2.9e-100 |
| CAD29794.1 | peptide\_synthetase | BGC0001015 | Microcystin\_biosynthetic\_gene\_cluster | 30 | 42.7027027027 | 366 | 2.9e-100 |
| ABX60153.1 | polyketide\_synthase | BGC0000978 | Cylindrospermopsin\_biosynthetic\_gene\_cluster | 30 | 42.6126126126 | 365 | 3.8e-100 |
| AHH99926.1 | PKS\_I | BGC0000002 | Aculeximycin\_biosynthetic\_gene\_cluster | 30 | 45.7207207207 | 365 | 3.8e-100 |
| CAO85893.1 | modular\_polyketide\_synthase\_NorA | BGC0000110 | Neoaureothin\_biosynthetic\_gene\_cluster | 28 | 51.6216216216 | 365 | 3.8e-100 |
| ABC84458.1 | NigAIII | BGC0000114 | Nigericin\_biosynthetic\_gene\_cluster | 28 | 47.4324324324 | 365 | 3.8e-100 |
| AEC13070.1 | fosD | BGC0000060 | Fostriecin\_biosynthetic\_gene\_cluster | 30 | 42.9279279279 | 365 | 3.8e-100 |
| BAK64650.1 | polyketide\_synthase | BGC0000135 | Reveromycin\_biosynthetic\_gene\_cluster | 29 | 46.0810810811 | 365 | 5e-100 |
| CAD55506.1 | putative\_type\_I\_polyketide\_synthase | BGC0000038 | Coelimycin\_biosynthetic\_gene\_cluster | 27 | 57.0720720721 | 365 | 5e-100 |
| AFP87523.1 | type\_I\_polyketide\_synthase | BGC0001159 | Marinopyrrole\_biosynthetic\_gene\_cluster | 30 | 42.972972973 | 364 | 6.6e-100 |
| AKD43753.1 | HerB | BGC0001349 | Heronamide\_biosynthetic\_gene\_cluster | 29 | 45.8108108108 | 364 | 6.6e-100 |
| BAF02926.1 | type\_I\_polyketide\_synthase | BGC0000073 | Halstoctacosanolide\_biosynthetic\_gene\_cluster | 29 | 46.3063063063 | 364 | 8.6e-100 |
| CAJ88186.1 | putative\_modular\_polyketide\_synthase | BGC0000151 | Stambomycin\_biosynthetic\_gene\_cluster | 27 | 55.6756756757 | 364 | 8.6e-100 |
| ALA09354.1 | type\_I\_modular\_PKS | BGC0001303 | Lobosamide\_biosynthetic\_gene\_cluster | 28 | 55.2252252252 | 364 | 8.6e-100 |
| AAF71775.1 | NysB | BGC0000115 | Nystatin\_biosynthetic\_gene\_cluster | 30 | 42.972972973 | 364 | 1.1e-99 |
| AGI99482.1 | Type\_I\_polyketide\_synthase | BGC0001004 | Lobophorin\_biosynthetic\_gene\_cluster | 29 | 46.7567567568 | 363 | 1.5e-99 |
| ABP55221.1 | acyl\_transferase\_domain\_protein | BGC0000142 | Salinilactam\_biosynthetic\_gene\_cluster | 31 | 41.8018018018 | 363 | 1.5e-99 |
| ACO94468.1 | polyketide\_synthase\_type\_I | BGC0000029 | BE-14106\_biosynthetic\_gene\_cluster | 28 | 50.1801801802 | 363 | 2.5e-99 |
| EGJ35088.1 | polyketide\_synthase\_module | BGC0001163 | 1-heptadecene\_biosynthetic\_gene\_cluster | 30 | 44.7747747748 | 363 | 2.5e-99 |
| ABC87511.1 | polyketide\_synthase | BGC0001011 | Meridamycin\_biosynthetic\_gene\_cluster | 30 | 42.7027027027 | 362 | 3.3e-99 |
| ADC45535.1 | modular\_polyketide\_synthase | BGC0000093 | Meilingmycin\_biosynthetic\_gene\_cluster | 29 | 47.7477477477 | 362 | 3.3e-99 |
| ctg1\_orf22 |  | BGC0001013 | Meridamycin\_biosynthetic\_gene\_cluster | 30 | 42.7027027027 | 362 | 3.3e-99 |
| AHH99923.1 | PKS\_I | BGC0000002 | Aculeximycin\_biosynthetic\_gene\_cluster | 30 | 43.6036036036 | 362 | 3.3e-99 |
| AAC69332.1 | type\_I\_polyketide\_synthase\_PikAIV | BGC0000094 | Methymycin\_/\_pikromycin\_biosynthetic\_gene\_cluster | 30 | 42.2972972973 | 362 | 4.2e-99 |
| AAQ82564.1 | FscC | BGC0000061 | FR-008\_biosynthetic\_gene\_cluster | 28 | 45.8558558559 | 362 | 4.2e-99 |
| CBW54671.1 | polyketide\_synthase/non\_ribosomal\_peptide\_synthetase | BGC0000971 | Cinnabaramide\_biosynthetic\_gene\_cluster | 31 | 41.8018018018 | 361 | 5.5e-99 |
| CBZ41585.1 | putative\_modular\_polyketide\_synthase | BGC0000151 | Stambomycin\_biosynthetic\_gene\_cluster | 28 | 45.8108108108 | 361 | 7.2e-99 |
| AGC24271.1 | PrlQ | BGC0001038 | Pyralomicin\_biosynthetic\_gene\_cluster | 31 | 39.5495495495 | 361 | 7.2e-99 |
| BAC68126.1 | modular\_polyketide\_synthase | BGC0000059 | Filipin\_biosynthetic\_gene\_cluster | 31 | 42.7927927928 | 361 | 9.5e-99 |
| AEC13069.1 | fosC | BGC0000060 | Fostriecin\_biosynthetic\_gene\_cluster | 27 | 58.8738738739 | 361 | 9.5e-99 |
| ADH04659.1 | TugC | BGC0001342 | Thuggacin\_biosynthetic\_gene\_cluster | 30 | 43.1081081081 | 360 | 1.2e-98 |
| AAG23266.1 | polyketide\_synthase\_extender\_modules\_3-4 | BGC0000148 | Spinosad\_biosynthetic\_gene\_cluster | 29 | 42.7927927928 | 360 | 1.6e-98 |
| BAC76491.1 | lankamycin\_synthase\_LkmAIII | BGC0000085 | Lankamycin\_biosynthetic\_gene\_cluster | 31 | 42.5225225225 | 360 | 1.6e-98 |
| AAX98185.1 | polyketide\_synthase\_type\_I | BGC0000052 | ECO-02301\_biosynthetic\_gene\_cluster | 30 | 42.7477477477 | 360 | 1.6e-98 |
| AJW65410.1 | type\_I\_modular\_polyketide\_synthase | BGC0001195 | Nocardiopsin\_biosynthetic\_gene\_cluster | 29 | 45.7207207207 | 359 | 3.6e-98 |
| ACN69992.1 | polyketide\_synthase | BGC0000079 | Indanomycin\_biosynthetic\_gene\_cluster | 30 | 44.3243243243 | 359 | 3.6e-98 |
| BAK64637.1 | polyketide\_synthase | BGC0000135 | Reveromycin\_biosynthetic\_gene\_cluster | 30 | 42.9279279279 | 359 | 3.6e-98 |
| BAQ21939.1 | putative\_type\_I\_polyketide\_synthase | BGC0001204 | Versipelostatin\_biosynthetic\_gene\_cluster | 31 | 43.018018018 | 358 | 4.7e-98 |
| AEP40939.1 | polyketide\_synthase\_type\_I | BGC0000021 | Apoptolidin\_biosynthetic\_gene\_cluster | 28 | 54.2342342342 | 358 | 6.1e-98 |
| AEU17898.1 | putative\_type\_I\_PKS | BGC0001072 | Simocyclinone\_biosynthetic\_gene\_cluster | 30 | 42.6126126126 | 358 | 6.1e-98 |
| ABY66019.1 | 6-methylsalicylic\_acid\_synthase | BGC0001008 | Maduropeptin\_biosynthetic\_gene\_cluster | 31 | 46.6666666667 | 357 | 1e-97 |
| ABP55493.1 | thioester\_reductase\_domain | BGC0001006 | Lymphostin\_biosynthetic\_gene\_cluster | 30 | 42.7927927928 | 357 | 1e-97 |
| ctg1\_orf28 |  | BGC0000096 | Midecamycin\_biosynthetic\_gene\_cluster | 28 | 46.5315315315 | 357 | 1e-97 |
| AHA38199.1 | GphF | BGC0000069 | Gephyronic\_acid\_biosynthetic\_gene\_cluster | 28 | 45.7207207207 | 357 | 1.4e-97 |
| ADH04660.1 | TugD | BGC0001342 | Thuggacin\_biosynthetic\_gene\_cluster | 30 | 43.1981981982 | 357 | 1.4e-97 |
| AAQ84156.1 | Plm1 | BGC0000123 | Phoslactomycin\_B\_biosynthetic\_gene\_cluster | 30 | 45.7657657658 | 356 | 1.8e-97 |
| BAF02925.1 | type\_I\_polyketide\_synthase | BGC0000073 | Halstoctacosanolide\_biosynthetic\_gene\_cluster | 29 | 46.2612612613 | 356 | 1.8e-97 |
| CAQ52626.1 | type\_I\_polyketide\_synthase,\_loading\_module\_and\_modules\_1-3 | BGC0001066 | Kendomycin\_biosynthetic\_gene\_cluster | 30 | 43.3783783784 | 356 | 3e-97 |
| AHE80996.1 | PieA6 | BGC0001169 | Piericidin\_A1\_biosynthetic\_gene\_cluster | 30 | 43.1981981982 | 356 | 3e-97 |
| CUW01176.1 | Type\_I\_polyketide\_synthase | BGC0001359\_c2 | PM100117\_/\_PM100118\_biosynthetic\_gene\_cluster | 26 | 55.4504504505 | 356 | 3e-97 |
| BAP34739.1 | type\_I\_polyketide\_synthase | BGC0000078 | Incednine\_biosynthetic\_gene\_cluster | 29 | 45.6756756757 | 356 | 3e-97 |
| CAA60459.1 | polyketide\_synthase | BGC0001040 | Rapamycin\_biosynthetic\_gene\_cluster | 30 | 45.6306306306 | 356 | 3e-97 |
| AAW03327.1 | CtaD | BGC0000982 | Cystothiazole\_A\_biosynthetic\_gene\_cluster | 28 | 54.3243243243 | 355 | 4e-97 |
| BAC68127.1 | modular\_polyketide\_synthase | BGC0000059 | Filipin\_biosynthetic\_gene\_cluster | 29 | 49.954954955 | 354 | 6.8e-97 |
| AGI99494.1 | Type\_I\_polyketide\_synthase | BGC0001004 | Lobophorin\_biosynthetic\_gene\_cluster | 30 | 42.972972973 | 354 | 6.8e-97 |
| AKD43761.1 | HerD | BGC0001349 | Heronamide\_biosynthetic\_gene\_cluster | 26 | 53.3333333333 | 354 | 6.8e-97 |
| CAJ88175.1 | putative\_polyketide\_synthase\_B | BGC0000151 | Stambomycin\_biosynthetic\_gene\_cluster | 29 | 42.5675675676 | 354 | 8.9e-97 |
| ACB37741.1 | putative\_type\_I\_polyketide\_synthase | BGC0000162 | Tetrocarcin\_A\_biosynthetic\_gene\_cluster | 28 | 54.954954955 | 354 | 8.9e-97 |
| CCP20047.1 | divK\_protein | BGC0001119 | Divergolide\_biosynthetic\_gene\_cluster | 29 | 43.7387387387 | 354 | 1.2e-96 |
| AAA79984.2 | soraphen\_polyketide\_synthase\_B | BGC0000147 | Soraphen\_biosynthetic\_gene\_cluster | 29 | 42.9279279279 | 354 | 1.2e-96 |
| CAQ34918.1 | nonribosomal\_peptide\_synthetase/\_polyketide\_synthase | BGC0000986 | Dkxanthene\_biosynthetic\_gene\_cluster | 26 | 59.5945945946 | 354 | 1.2e-96 |
| AEH42474.1 | polyketide\_synthase | BGC0000032 | Calcimycin\_biosynthetic\_gene\_cluster | 28 | 44.5045045045 | 354 | 1.2e-96 |
| AAG23264.1 | polyketide\_synthase\_loading\_and\_extender\_module\_1 | BGC0000148 | Spinosad\_biosynthetic\_gene\_cluster | 30 | 42.6576576577 | 353 | 1.5e-96 |
| BAR73021.1 | putative\_PKS\_(KS-AT-KR-ACP) | BGC0001194 | Hitachimycin\_biosynthetic\_gene\_cluster | 30 | 42.3423423423 | 353 | 1.5e-96 |
| ABK32259.1 | AmbE | BGC0000014 | Ambruticin\_biosynthetic\_gene\_cluster | 29 | 46.0810810811 | 353 | 1.5e-96 |
| AFL48525.1 | laidlomycin\_polyketide\_synthase\_(loading\_module\_and\_module\_1) | BGC0000084 | Laidlomycin\_biosynthetic\_gene\_cluster | 30 | 42.7477477477 | 353 | 1.5e-96 |
| ADH04658.1 | TugB | BGC0001342 | Thuggacin\_biosynthetic\_gene\_cluster | 29 | 43.1981981982 | 352 | 2.6e-96 |
| AAC38075.1 | polyketide\_synthase\_type\_I | BGC0000127 | Pyoluteorin\_biosynthetic\_gene\_cluster | 30 | 39.5045045045 | 352 | 2.6e-96 |
| CAL58683.1 | polyketide\_synthase | BGC0000149 | Spirangien\_biosynthetic\_gene\_cluster | 29 | 42.972972973 | 352 | 2.6e-96 |
| BAG84248.1 | putative\_polyketide\_synthase | BGC0000257 | Prodigiosin\_biosynthetic\_gene\_cluster | 30 | 41.8018018018 | 352 | 2.6e-96 |
| AGC09487.1 | LobS5 | BGC0001183 | Lobophorin\_biosynthetic\_gene\_cluster | 29 | 43.7387387387 | 352 | 3.4e-96 |
| CUW01171.1 | Type\_I\_polyketide\_synthase | BGC0001359 | PM100117\_/\_PM100118\_biosynthetic\_gene\_cluster | 29 | 43.0630630631 | 352 | 4.4e-96 |
| ACY06290.1 | type\_I\_polyketide\_synthase | BGC0001042 | Sanglifehrin\_A\_biosynthetic\_gene\_cluster | 29 | 43.2432432432 | 351 | 7.5e-96 |
| EAU32819.1 | 6-methylsalicylic\_acid\_synthase | BGC0000160 | Terreic\_acid\_biosynthetic\_gene\_cluster | 28 | 47.8828828829 | 351 | 9.8e-96 |
| BAA20102.2 | 6-methylsalicylic\_acid\_synthase | BGC0001276 | 6-methylsalicyclic\_acid\_biosynthetic\_gene\_cluster | 28 | 47.7027027027 | 350 | 1.3e-95 |
| AAP42858.1 | NanA4 | BGC0000105 | Nanchangmycin\_biosynthetic\_gene\_cluster | 27 | 46.3513513514 | 350 | 1.7e-95 |
| AAQ82561.1 | FscA | BGC0000061 | FR-008\_biosynthetic\_gene\_cluster | 29 | 43.6036036036 | 350 | 1.7e-95 |
| CAL58684.1 | polyketide\_synthase | BGC0000149 | Spirangien\_biosynthetic\_gene\_cluster | 29 | 43.1981981982 | 349 | 2.2e-95 |
| ABB05103.1 | LipPks2 | BGC0001003 | Lipomycin\_biosynthetic\_gene\_cluster | 28 | 48.6936936937 | 349 | 2.2e-95 |
| CAD89775.1 | MelD\_protein | BGC0001010 | Melithiazol\_biosynthetic\_gene\_cluster | 27 | 55.1351351351 | 349 | 2.2e-95 |
| AEZ53947.1 | polyketide\_synthase | BGC0000144 | Salinomycin\_biosynthetic\_gene\_cluster | 29 | 42.7027027027 | 348 | 4.9e-95 |
| BAE93722.1 | type\_I\_polyketide\_synthase | BGC0000164 | Tetronomycin\_biosynthetic\_gene\_cluster | 28 | 46.2612612613 | 348 | 4.9e-95 |
| CAJ88184.1 | putative\_modular\_polyketide\_synthase | BGC0000151 | Stambomycin\_biosynthetic\_gene\_cluster | 30 | 42.2072072072 | 348 | 4.9e-95 |
| AAQ84144.1 | Plm4 | BGC0000123 | Phoslactomycin\_B\_biosynthetic\_gene\_cluster | 30 | 42.027027027 | 348 | 6.3e-95 |
| AEZ53949.1 | polyketide\_synthase | BGC0000144 | Salinomycin\_biosynthetic\_gene\_cluster | 29 | 43.1981981982 | 348 | 6.3e-95 |
| AAM54076.1 | polyketide\_synthase | BGC0000020 | Ansamitocin\_biosynthetic\_gene\_cluster | 30 | 43.3333333333 | 348 | 6.3e-95 |
| BAF85838.1 | modular\_polyketide\_synthase | BGC0000109 | Nemadectin\_biosynthetic\_gene\_cluster | 30 | 43.1081081081 | 348 | 6.3e-95 |
| EYT83439.1 | beta-ketoacyl\_synthase | BGC0001213 | Nataxazole\_biosynthetic\_gene\_cluster | 30 | 43.4684684685 | 347 | 8.3e-95 |
| AEZ53945.1 | polyketide\_synthase | BGC0000144 | Salinomycin\_biosynthetic\_gene\_cluster | 29 | 46.036036036 | 347 | 8.3e-95 |
| ACO94488.1 | polyketide\_synthase\_type\_I | BGC0000097 | ML-449\_biosynthetic\_gene\_cluster | 29 | 48.4684684685 | 347 | 8.3e-95 |
| BI06\_RS39060 | polyketide\_synthase | BGC0001232 | Leucanicidin\_biosynthetic\_gene\_cluster | 31 | 43.1081081081 | 347 | 1.1e-94 |
| ACB37743.1 | putative\_type\_I\_polyketide\_synthase | BGC0000162 | Tetrocarcin\_A\_biosynthetic\_gene\_cluster | 29 | 43.1081081081 | 347 | 1.1e-94 |
| ACO94499.1 | polyketide\_synthase\_type\_I | BGC0000097 | ML-449\_biosynthetic\_gene\_cluster | 28 | 46.1711711712 | 346 | 1.8e-94 |
| ctg1\_orf521 |  | BGC0001199 | Akaeolide\_biosynthetic\_gene\_cluster | 29 | 42.5225225225 | 346 | 1.8e-94 |
| CAQ43075.1 | polyketide\_synthase | BGC0000970 | Chondrochloren\_biosynthetic\_gene\_cluster | 31 | 36.8468468468 | 345 | 4.1e-94 |
| ACY13415.1 | KR\_domain\_protein | BGC0001367 | Haliamide\_biosynthetic\_gene\_cluster | 30 | 42.5225225225 | 345 | 4.1e-94 |
| ABK32258.1 | AmbD | BGC0000014 | Ambruticin\_biosynthetic\_gene\_cluster | 30 | 46.3963963964 | 345 | 4.1e-94 |
| ADX66470.1 | ScnS0 | BGC0000108 | Natamycin\_biosynthetic\_gene\_cluster | 29 | 43.2432432432 | 345 | 5.4e-94 |
| AHH25595.1 | PKS | BGC0000957 | Ansatrienin\_(mycotrienin)\_biosynthetic\_gene\_cluster | 28 | 46.1261261261 | 344 | 7e-94 |
| AGC24270.1 | PrlP | BGC0001038 | Pyralomicin\_biosynthetic\_gene\_cluster | 30 | 42.4774774775 | 344 | 7e-94 |
| AAF71776.1 | NysC | BGC0000115 | Nystatin\_biosynthetic\_gene\_cluster | 28 | 45.7207207207 | 343 | 1.6e-93 |
| AFP87524.1 | type\_I\_polyketide\_synthase | BGC0001159 | Marinopyrrole\_biosynthetic\_gene\_cluster | 31 | 41.6666666667 | 342 | 2.7e-93 |
| AGC45624.1 | polyketide\_synthase | BGC0001394 | Phenalamide\_biosynthetic\_gene\_cluster | 31 | 36.1261261261 | 342 | 2.7e-93 |
| ABB05102.1 | LipPks1 | BGC0001003 | Lipomycin\_biosynthetic\_gene\_cluster | 29 | 43.6486486486 | 342 | 2.7e-93 |
| AAP42859.1 | NanA5 | BGC0000105 | Nanchangmycin\_biosynthetic\_gene\_cluster | 28 | 47.0720720721 | 342 | 3.5e-93 |
| AFI57005.1 | QmnA1 | BGC0000133 | Quartromicin\_biosynthetic\_gene\_cluster | 26 | 61.2612612613 | 342 | 4.5e-93 |
| ACY06288.1 | type\_I\_polyketide\_synthase | BGC0001042 | Sanglifehrin\_A\_biosynthetic\_gene\_cluster | 30 | 42.7927927928 | 341 | 5.9e-93 |
| AHH99924.1 | PKS\_I | BGC0000002 | Aculeximycin\_biosynthetic\_gene\_cluster | 29 | 45.9009009009 | 340 | 1.7e-92 |
| ACF35447.1 | MbcAIII | BGC0000090 | Macbecin\_biosynthetic\_gene\_cluster | 28 | 45.7207207207 | 339 | 2.3e-92 |
| CBA11584.1 | polyketide\_synthase\_type\_I | BGC0001046 | Streptolydigin\_biosynthetic\_gene\_cluster | 28 | 44.4594594595 | 339 | 2.3e-92 |
| CCE88378.1 | polyketide\_synthase | BGC0001034 | Pellasoren\_biosynthetic\_gene\_cluster | 30 | 36.2162162162 | 339 | 2.3e-92 |
| WP\_043476519.1 | polyketide\_synthase | BGC0001232 | Leucanicidin\_biosynthetic\_gene\_cluster | 30 | 43.0630630631 | 339 | 2.9e-92 |
| CAJ88185.2 | putative\_modular\_polyketide\_synthase | BGC0000151 | Stambomycin\_biosynthetic\_gene\_cluster | 27 | 46.1711711712 | 338 | 5e-92 |
| CAM00065.1 | EryAIII\_Erythromycin\_polyketide\_synthase\_modules\_5\_and\_6 | BGC0000055 | Erythromycin\_biosynthetic\_gene\_cluster | 29 | 42.2072072072 | 338 | 5e-92 |
| AAK73501.1 | AmphI | BGC0000015 | Amphotericin\_biosynthetic\_gene\_cluster | 29 | 42.7027027027 | 338 | 6.6e-92 |
| ADC79617.1 | BafAII | BGC0000028 | Bafilomycin\_biosynthetic\_gene\_cluster | 30 | 42.7927927928 | 337 | 8.6e-92 |
| ACO94460.1 | polyketide\_synthase\_type\_I | BGC0000029 | BE-14106\_biosynthetic\_gene\_cluster | 29 | 48.2882882883 | 336 | 1.9e-91 |
| CAE45668.1 | borrelidin\_polyketide\_synthase,\_type\_I | BGC0000031 | Borrelidin\_biosynthetic\_gene\_cluster | 29 | 45.0900900901 | 336 | 3.3e-91 |
| CUI25702.1 | Tu3010\_Hybrid\_PKS-NRPS\_system | BGC0001352 | Thiotetronate\_Tu\_3010\_biosynthetic\_gene\_cluster | 27 | 48.4234234234 | 336 | 3.3e-91 |
| AGI99496.1 | Type\_I\_polyketide\_synthase | BGC0001004 | Lobophorin\_biosynthetic\_gene\_cluster | 27 | 56.7117117117 | 334 | 7.3e-91 |
| CQR60493.1 | Polyketide\_synthase,\_type\_I,\_modules:\_9\_and\_10 | BGC0001287 | Chaxamycin\_A\_/\_chaxamycin\_B\_/\_chaxamycin\_C\_/\_chaxamycin\_D | 27 | 56.3513513514 | 334 | 7.3e-91 |
| ADM46357.1 | polyketide\_synthase | BGC0000106 | Naphthomycin\_biosynthetic\_gene\_cluster | 29 | 46.3963963964 | 334 | 9.5e-91 |
| CCE88376.1 | polyketide\_synthase | BGC0001034 | Pellasoren\_biosynthetic\_gene\_cluster | 33 | 29.7297297297 | 334 | 1.2e-90 |
| AAZ77673.1 | ChlB1 | BGC0000036 | Chlorothricin\_biosynthetic\_gene\_cluster | 31 | 39.8648648649 | 333 | 2.1e-90 |
| ACA99172.1 | Polyketide\_synthase | BGC0001160 | Nonadecene\_biosynthetic\_gene\_cluster | 31 | 42.4324324324 | 332 | 3.6e-90 |
| AAG13917.1 | megalomicin\_6-deoxyerythronolide\_B\_synthase\_1 | BGC0000092 | Megalomicin\_biosynthetic\_gene\_cluster | 27 | 46.3063063063 | 332 | 3.6e-90 |
| AAU93806.2 | polyketide\_synthase\_modules\_3\_and\_4 | BGC0000054 | Erythromycin\_biosynthetic\_gene\_cluster | 28 | 46.1261261261 | 332 | 3.6e-90 |
| AAQ90174.1 | polyketide\_synthase\_type\_I | BGC0000128 | Pyoluteorin\_biosynthetic\_gene\_cluster | 29 | 42.3423423423 | 332 | 4.7e-90 |
| ABW96541.1 | type\_I\_modular\_polyketide\_synthase | BGC0000159 | Tautomycin\_biosynthetic\_gene\_cluster | 29 | 43.0630630631 | 331 | 1e-89 |
| CAE45669.1 | borrelidin\_polyketide\_synthase,\_type\_I | BGC0000031 | Borrelidin\_biosynthetic\_gene\_cluster | 27 | 46.3513513514 | 329 | 2.3e-89 |
| CBZ41586.1 | putative\_modular\_polyketide\_synthase | BGC0000151 | Stambomycin\_biosynthetic\_gene\_cluster | 29 | 42.4324324324 | 329 | 4e-89 |
| CBA11582.1 | polyketide\_synthase\_type\_I | BGC0001046 | Streptolydigin\_biosynthetic\_gene\_cluster | 27 | 46.4414414414 | 329 | 4e-89 |
| BAD38874.1 | polyketide\_synthase | BGC0000111 | Neocarzilin\_biosynthetic\_gene\_cluster | 30 | 38.8738738739 | 329 | 4e-89 |
| CAL58687.1 | polyketide\_synthase | BGC0000149 | Spirangien\_biosynthetic\_gene\_cluster | 29 | 43.2882882883 | 327 | 1.2e-88 |
| BAB69193.1 |  | BGC0000117 | Oligomycin\_biosynthetic\_gene\_cluster | 29 | 42.4324324324 | 327 | 1.2e-88 |
| AIW00670.1 | mellein\_synthase | BGC0001244 | (-)-Mellein\_biosynthetic\_gene\_cluster | 26 | 49.1441441441 | 327 | 1.5e-88 |
| AAF62883.1 | epoD | BGC0000991 | Epothilone\_biosynthetic\_gene\_cluster | 31 | 35.4954954955 | 326 | 2e-88 |
| ADB12491.1 | EpoD | BGC0000990 | Epothilone\_biosynthetic\_gene\_cluster | 31 | 35.4954954955 | 326 | 2.6e-88 |
| ABM21569.1 | CrpA | BGC0000975 | Cryptophycin\_biosynthetic\_gene\_cluster | 27 | 48.8288288288 | 326 | 2.6e-88 |
| ADC45534.1 | modular\_polyketide\_synthase | BGC0000093 | Meilingmycin\_biosynthetic\_gene\_cluster | 29 | 43.2882882883 | 326 | 3.4e-88 |
| ABW96542.1 | type\_I\_modular\_polyketide\_synthase | BGC0000159 | Tautomycin\_biosynthetic\_gene\_cluster | 28 | 42.7027027027 | 325 | 4.4e-88 |
| ADC79619.1 | BafAIV | BGC0000028 | Bafilomycin\_biosynthetic\_gene\_cluster | 29 | 43.6486486486 | 325 | 5.8e-88 |
| EJK79843.1 | amino\_acid\_adenylation\_enzyme/thioester\_reductase\_family\_protein | BGC0000436 | Syringolin\_A\_biosynthetic\_gene\_cluster | 28 | 49.9099099099 | 325 | 5.8e-88 |
| ABG02264.1 | SalB | BGC0000143 | Salinomycin\_biosynthetic\_gene\_cluster | 28 | 42.7477477477 | 324 | 9.8e-88 |
| ACR50782.1 | polyketide\_synthase | BGC0000163 | Tetronasin\_biosynthetic\_gene\_cluster | 28 | 43.0630630631 | 324 | 9.8e-88 |
| ADU86002.1 | putative\_modular\_polyketide\_synthase | BGC0000165 | Tiacumicin\_B\_biosynthetic\_gene\_cluster | 29 | 42.2972972973 | 324 | 9.8e-88 |
| ctg1\_orf7 |  | BGC0000053 | Elaiophylin\_biosynthetic\_gene\_cluster | 29 | 42.8828828829 | 323 | 1.7e-87 |
| ABY21542.1 | AngAV | BGC0000018 | Angolamycin\_biosynthetic\_gene\_cluster | 29 | 43.5135135135 | 323 | 2.2e-87 |
| ABB88519.1 | polyketide\_synthase\_type\_I | BGC0000050 | E-837\_biosynthetic\_gene\_cluster | 30 | 43.4234234234 | 323 | 2.2e-87 |
| AAQ84147.1 | Plm7 | BGC0000123 | Phoslactomycin\_B\_biosynthetic\_gene\_cluster | 29 | 42.2522522523 | 322 | 2.9e-87 |
| AGI61658.1 | modular\_PKS | BGC0000095\_c3 | Micromonolactam\_biosynthetic\_gene\_cluster | 27 | 46.3063063063 | 322 | 3.7e-87 |
| ABI91470.1 | beta-ketoacyl\_synthase | BGC0001094 | Enacyloxin\_biosynthetic\_gene\_cluster | 29 | 47.7027027027 | 322 | 4.9e-87 |
| CAE45672.1 | Borrelidin\_polyketide\_synthase,\_type\_I | BGC0000031 | Borrelidin\_biosynthetic\_gene\_cluster | 28 | 42.7927927928 | 322 | 4.9e-87 |
| BAB69192.1 | modular\_polyketide\_synthase | BGC0000117 | Oligomycin\_biosynthetic\_gene\_cluster | 29 | 40.4054054054 | 321 | 8.3e-87 |
| AKN45693.1 | polyketide\_synthase | BGC0001284 | Alternariol\_biosynthetic\_gene\_cluster | 28 | 44.0990990991 | 320 | 1.9e-86 |
| ABB88535.1 | polyketide\_synthase\_type\_I | BGC0000049\_c2 | E-492\_/\_E-975\_biosynthetic\_gene\_cluster | 27 | 46.8018018018 | 319 | 2.4e-86 |
| AAR87760.2 | ZmaK | BGC0001059 | Zwittermycin\_A\_biosynthetic\_gene\_cluster | 27 | 42.3873873874 | 315 | 4.6e-85 |
| ADM46358.1 | polyketide\_synthase | BGC0000106 | Naphthomycin\_biosynthetic\_gene\_cluster | 26 | 55.5855855856 | 315 | 6e-85 |
| AAC46024.1 | polyketide\_synthase\_modules\_1\_and\_2 | BGC0000113 | Niddamycin\_biosynthetic\_gene\_cluster | 29 | 42.3423423423 | 314 | 7.8e-85 |
| ADI24926.1 | VrtA | BGC0000168 | Viridicatumtoxin\_biosynthetic\_gene\_cluster | 28 | 42.8378378378 | 314 | 1e-84 |
| ADC45516.1 | modular\_polyketide\_synthase | BGC0000093 | Meilingmycin\_biosynthetic\_gene\_cluster | 29 | 42.2522522523 | 313 | 1.7e-84 |
| AHH99921.1 | PKS\_I | BGC0000002 | Aculeximycin\_biosynthetic\_gene\_cluster | 29 | 42.4324324324 | 311 | 6.6e-84 |
| CCA29203.1 | non-ribosomal\_peptide\_synthetase/polyketide\_synthase | BGC0000955 | Althiomycin\_biosynthetic\_gene\_cluster | 28 | 42.7477477477 | 310 | 1.5e-83 |
| AEE88283.1 | CurG | BGC0000976 | Curacin\_biosynthetic\_gene\_cluster | 28 | 42.7927927928 | 309 | 2.5e-83 |
| AAT70102.1 | CurG | BGC0001165 | Curacin\_biosynthetic\_gene\_cluster | 28 | 42.7927927928 | 309 | 2.5e-83 |
| BAQ25511.1 | type\_I\_polyketide\_synthase | BGC0001288 | Maklamicin\_biosynthetic\_gene\_cluster | 32 | 30.990990991 | 309 | 3.3e-83 |
| AAP42857.1 | NanA3 | BGC0000105 | Nanchangmycin\_biosynthetic\_gene\_cluster | 29 | 40.4504504505 | 307 | 9.5e-83 |
| CAL80821.1 | sylD-like\_NRPS/PKS | BGC0000997 | Glidobactin\_biosynthetic\_gene\_cluster | 29 | 43.1081081081 | 306 | 2.8e-82 |
| AJO72743.1 | Type\_I\_modular\_polyketide\_synthase | BGC0001381 | Brasilinolide\_biosynthetic\_gene\_cluster | 29 | 42.5225225225 | 305 | 4.7e-82 |
| AJO72734.1 | Type\_I\_modular\_polyketide\_synthase | BGC0001381 | Brasilinolide\_biosynthetic\_gene\_cluster | 27 | 43.8738738739 | 303 | 2.3e-81 |
| AAN32979.1 | BarE | BGC0000962 | Barbamide\_biosynthetic\_gene\_cluster | 29 | 44.7297297297 | 302 | 3.1e-81 |
| CAJ76291.1 | putative\_polyketide\_synthase | BGC0000972 | Colibactin\_biosynthetic\_gene\_cluster | 29 | 43.3783783784 | 302 | 5.2e-81 |
| AHB82072.1 | non\_ribosomal\_peptide\_synthetase/polyketide\_synthase | BGC0001231 | Microsclerodermins\_biosynthetic\_gene\_cluster | 29 | 43.8288288288 | 301 | 8.9e-81 |
| EGD99348.1 | polyketide\_synthase | BGC0001144 | Neosartoricin\_biosynthetic\_gene\_cluster | 29 | 42.4774774775 | 300 | 1.2e-80 |
| AAU93805.2 | polyketide\_synthase\_modules\_5\_and\_6 | BGC0000054 | Erythromycin\_biosynthetic\_gene\_cluster | 28 | 39.8198198198 | 300 | 1.5e-80 |
| EAU38791.1 | hypothetical\_protein | BGC0000161 | Terrein\_biosynthetic\_gene\_cluster | 27 | 42.7477477477 | 300 | 1.5e-80 |
| BAE93729.1 | type\_I\_polyketide\_synthase | BGC0000164 | Tetronomycin\_biosynthetic\_gene\_cluster | 27 | 43.1981981982 | 300 | 1.5e-80 |
| AGC09485.1 | LobS2 | BGC0001183 | Lobophorin\_biosynthetic\_gene\_cluster | 26 | 55.9009009009 | 299 | 4.4e-80 |
| AHV78253.1 | ResS2 | BGC0001246 | Trans-resorcylide\_biosynthetic\_gene\_cluster | 29 | 43.8288288288 | 295 | 3.7e-79 |
| CAD70195.1 | non-ribosomal\_peptide\_synthetase | BGC0001047 | Syringolin\_A\_biosynthetic\_gene\_cluster | 27 | 42.8378378378 | 295 | 3.7e-79 |
| AEK75504.1 | type\_1\_polyketide\_synthase | BGC0000001 | Abyssomicin\_biosynthetic\_gene\_cluster | 29 | 42.7027027027 | 295 | 3.7e-79 |
| ACH72912.1 | AflC | BGC0000011 | Aflatoxin/sterigmatocystin\_biosynthetic\_gene\_cluster | 28 | 42.7477477477 | 295 | 4.9e-79 |
| ABU63483.1 | polyketide\_synthase | BGC0001272 | Elsinochrome\_B\_/\_elsinochrome\_C\_biosynthetic\_gene\_cluster | 28 | 43.5135135135 | 295 | 6.4e-79 |
| ADZ24995.1 | non-ribosomal\_peptide\_synthase/polyketide\_synthase | BGC0000380 | Leupyrrin\_biosynthetic\_gene\_cluster | 26 | 56.2612612613 | 292 | 4.1e-78 |
| WP\_042799407.1 | polyketide\_synthase | BGC0001283 | Arsenopolyketides\_biosynthetic\_gene\_cluster | 34 | 24.8648648649 | 292 | 4.1e-78 |
| AAS90047.1 | PksA | BGC0000009 | Aflatoxin\_biosynthetic\_gene\_cluster | 28 | 42.6126126126 | 291 | 7e-78 |
| AHD05614.1 | putative\_non-ribosomal\_peptide\_ligase/\_polyketide\_synthase\_hybrid | BGC0001033 | Paenilamicin\_biosynthetic\_gene\_cluster | 28 | 42.972972973 | 291 | 9.2e-78 |
| AAS89999.1 | PksA | BGC0000007 | Aflatoxin\_biosynthetic\_gene\_cluster | 28 | 42.972972973 | 290 | 2.1e-77 |
| CCT67991.1 | bikaverin\_cluster-polyketide\_synthase | BGC0000030 | Bikaverin\_biosynthetic\_gene\_cluster | 28 | 43.6036036036 | 289 | 3.5e-77 |
| CAA73127.1 | HMWP1\_protein | BGC0000467 | Yersiniabactin\_biosynthetic\_gene\_cluster | 28 | 42.7477477477 | 289 | 4.6e-77 |
| ACM79805.1 | ZmaA | BGC0001059 | Zwittermycin\_A\_biosynthetic\_gene\_cluster | 27 | 42.3423423423 | 288 | 7.8e-77 |
| WP\_048832936.1 | polyketide\_synthase | BGC0001348 | JBIR-100\_biosynthetic\_gene\_cluster | 31 | 30.5855855856 | 287 | 1e-76 |
| AAS90022.1 | PksA | BGC0000008 | Aflatoxin\_biosynthetic\_gene\_cluster | 28 | 42.9279279279 | 285 | 5.1e-76 |
| ABX37384.1 | Beta-ketoacyl\_synthase | BGC0000984 | Delftibactin\_biosynthetic\_gene\_cluster | 28 | 43.6036036036 | 285 | 6.6e-76 |
| AHH99922.1 | PKS\_I | BGC0000002 | Aculeximycin\_biosynthetic\_gene\_cluster | 28 | 42.8828828829 | 285 | 6.6e-76 |
| CAO98849.1 | polyketide\_synthase\_AufF | BGC0000023 | Aurafuron\_biosynthetic\_gene\_cluster | 35 | 24.6396396396 | 284 | 8.6e-76 |
| AAS90093.1 | PksA | BGC0000006 | Aflatoxin\_biosynthetic\_gene\_cluster | 28 | 42.9279279279 | 284 | 1.5e-75 |
| EAL84397.1 | polyketide\_synthase,\_putative | BGC0001118 | Endocrocin\_biosynthetic\_gene\_cluster | 27 | 42.8828828829 | 280 | 2.1e-74 |
| AEZ54375.1 | PieA2 | BGC0000124 | Piericidin\_A1\_biosynthetic\_gene\_cluster | 35 | 20.1801801802 | 279 | 2.8e-74 |
| ERM18799.1 | polyketide\_synthase | BGC0000172 | Basiliskamides\_biosynthetic\_gene\_cluster | 34 | 24.8648648649 | 279 | 2.8e-74 |
| EED53479.1 | polyketide\_synthase,\_putative | BGC0001304 | Aflavarin\_biosynthetic\_gene\_cluster | 27 | 42.8828828829 | 279 | 4.7e-74 |
| ACY01400.1 | AT-less\_polyketide\_synthase | BGC0000083 | Lactimidomycin\_biosynthetic\_gene\_cluster | 34 | 24.7747747748 | 278 | 6.2e-74 |
| BAV19379.1 | polyketide\_synthase | BGC0001390 | LL-Z1272beta\_biosynthetic\_gene\_cluster | 28 | 34.954954955 | 277 | 1.8e-73 |
| AAF00957.1 | McyG | BGC0001017 | Microcystin\_biosynthetic\_gene\_cluster | 28 | 43.5585585586 | 276 | 3.1e-73 |
| AMH40443.1 | PKS | BGC0001350 | Phormidolide\_biosynthetic\_gene\_cluster | 35 | 24.8648648649 | 275 | 5.2e-73 |
| ABV83230.1 | CppC | BGC0000116 | Nystatin-like\_Pseudonocardia\_polyene\_biosynthetic\_gene\_cluster | 31 | 29.7747747748 | 275 | 6.8e-73 |
| CAE46851.1 | Type\_I\_modular\_polyketide\_synthase | BGC0000103 | Mycolactone\_biosynthetic\_gene\_cluster | 36 | 24.0990990991 | 274 | 1.5e-72 |
| AAO62585.1 | peptide\_sythetase\_polyketide\_synthase\_fusion\_protein | BGC0001016 | Microcystin\_biosynthetic\_gene\_cluster | 28 | 43.1981981982 | 274 | 1.5e-72 |
| CAE46843.1 | Type\_I\_modular\_polyketide\_synthase | BGC0000103 | Mycolactone\_biosynthetic\_gene\_cluster | 36 | 24.0990990991 | 274 | 1.5e-72 |
| AGY62754.1 | EbeB | BGC0000051 | Ebelactone\_biosynthetic\_gene\_cluster | 30 | 35.9459459459 | 273 | 2e-72 |
| CBF70387.1 | polyketide\_synthase,\_putative\_(JCVI) | BGC0000684 | Asperthecin\_biosynthetic\_gene\_cluster | 27 | 42.4324324324 | 272 | 3.4e-72 |
| CAM00062.1 | EryAI\_Erythromycin\_polyketide\_synthase\_modules\_1\_and\_2 | BGC0000055 | Erythromycin\_biosynthetic\_gene\_cluster | 35 | 20.1801801802 | 271 | 7.5e-72 |
| ACN69988.1 | polyketide\_synthase | BGC0000079 | Indanomycin\_biosynthetic\_gene\_cluster | 36 | 20.045045045 | 269 | 2.9e-71 |
| ABS90475.1 | PKS | BGC0001106 | Oxazolomycin\_biosynthetic\_gene\_cluster | 37 | 20.0900900901 | 269 | 4.9e-71 |
| AJW65408.1 | type\_I\_modular\_polyketide\_synthase | BGC0001195 | Nocardiopsin\_biosynthetic\_gene\_cluster | 31 | 29.4594594595 | 267 | 1.4e-70 |
| CCH26295.1 | putative\_polyketide\_synthase | BGC0001158\_c6 | Bikaverin\_biosynthetic\_gene\_cluster | 28 | 43.3783783784 | 267 | 1.9e-70 |
| ABM63527.1 | BryB | BGC0000174 | Bryostatin\_biosynthetic\_gene\_cluster | 34 | 25.2702702703 | 265 | 7.1e-70 |
| ABS90470.1 | NRPS/PKS | BGC0001106 | Oxazolomycin\_biosynthetic\_gene\_cluster | 36 | 20.3153153153 | 264 | 1.6e-69 |
| ALP32043.1 | CycC | BGC0001293 | Cyclizidine\_biosynthetic\_gene\_cluster | 28 | 36.8468468468 | 263 | 2.1e-69 |
| AIJ04680.1 | polyketide\_synthase | BGC0001383 | Macrolactin\_biosynthetic\_gene\_cluster | 33 | 23.018018018 | 263 | 2.7e-69 |
| ABC34176.1 | polyketide\_synthase,\_putative | BGC0000186 | Thailandamide\_biosynthetic\_gene\_cluster | 34 | 25.0900900901 | 262 | 3.5e-69 |
| CAG23965.1 | polyketide\_synthase\_type\_I | BGC0000181 | Macrolactin\_biosynthetic\_gene\_cluster | 33 | 23.018018018 | 262 | 3.5e-69 |
| CBJ89760.1 | Polyketide\_synthase\_involved\_in\_xenocoumacin\_synthesis | BGC0001054 | Xenocoumacin\_biosynthetic\_gene\_cluster | 26 | 42.7927927928 | 262 | 6e-69 |
| AHA38200.1 | GphG | BGC0000069 | Gephyronic\_acid\_biosynthetic\_gene\_cluster | 32 | 29.5945945946 | 261 | 1e-68 |
| ABC33986.1 | polyketide\_synthase,\_putative | BGC0000186 | Thailandamide\_biosynthetic\_gene\_cluster | 32 | 24.4144144144 | 260 | 1.3e-68 |
| CAN93347.1 | Polyketide\_synthase | BGC0000179 | Etnangien\_biosynthetic\_gene\_cluster | 32 | 25.1351351351 | 260 | 1.7e-68 |
| AAS79461.1 | polyketide\_synthase\_subunit | BGC0000035 | Chalcomycin\_biosynthetic\_gene\_cluster | 32 | 25.9009009009 | 260 | 1.7e-68 |
| CTQ34881.1 | AtcD;\_polyketide\_synthase,\_modules\_1-4 | BGC0001301 | Anthracimycin\_biosynthetic\_gene\_cluster | 35 | 20.2252252252 | 258 | 6.6e-68 |
| ABS90471.1 | PKS\_type\_I | BGC0001106 | Oxazolomycin\_biosynthetic\_gene\_cluster | 32 | 25.990990991 | 256 | 2.5e-67 |
| AAD38786.1 | polyketide\_synthase | BGC0001257 | 1,3,6,8-tetrahydroxynaphthalene\_biosynthetic\_gene\_cluster | 27 | 42.7027027027 | 256 | 2.5e-67 |
| AFX60336.1 | polyketide\_synthase | BGC0001032 | Oocydin\_A\_biosynthetic\_gene\_cluster | 33 | 25.1801801802 | 256 | 3.3e-67 |
| AAP42873.1 | NanA11 | BGC0000105 | Nanchangmycin\_biosynthetic\_gene\_cluster | 31 | 28.1981981982 | 254 | 9.5e-67 |
| ABY21540.1 | AngAIII | BGC0000018 | Angolamycin\_biosynthetic\_gene\_cluster | 31 | 25.9459459459 | 254 | 9.5e-67 |
| AFN27483.1 | pks\_BonD | BGC0000173 | Bongkrekic\_acid\_biosynthetic\_gene\_cluster | 38 | 20.0900900901 | 253 | 1.6e-66 |
| CAE14178.1 |  | BGC0001180 | Isopropylstilbene\_biosynthetic\_gene\_cluster | 33 | 23.4684684685 | 253 | 2.1e-66 |
| CBF80428.1 | polyketide\_synthase,\_putative\_(JCVI) | BGC0000680 | Austinol/dehydroaustinol,\_locus\_1\_biosynthetic\_gene\_cluster | 25 | 43.4234234234 | 252 | 6.2e-66 |
| AAF71767.1 | NysJ | BGC0000115 | Nystatin\_biosynthetic\_gene\_cluster | 33 | 23.5135135135 | 252 | 6.2e-66 |
| CAL69890.1 | RhiC\_protein | BGC0001112 | Rhizoxins\_biosynthetic\_gene\_cluster | 35 | 20.5855855856 | 250 | 1.4e-65 |
| ACR50791.1 | putative\_polyketide\_synthase | BGC0000163 | Tetronasin\_biosynthetic\_gene\_cluster | 33 | 23.7837837838 | 249 | 3.1e-65 |
| CCE67070.1 | polyketide\_synthase | BGC0001242 | Fusarubin\_biosynthetic\_gene\_cluster | 26 | 43.6936936937 | 249 | 4e-65 |
| ctg1\_orf253 |  | BGC0001200 | Lorneic\_acid\_A\_biosynthetic\_gene\_cluster | 31 | 29.7747747748 | 247 | 1.2e-64 |
| ABC34675.1 | polyketide\_synthase,\_putative | BGC0000186 | Thailandamide\_biosynthetic\_gene\_cluster | 36 | 20.6756756757 | 247 | 1.2e-64 |
| ADA69239.2 | trans-AT\_hybrid\_polyketide\_synthase-NRPS | BGC0001071 | Nosperin\_biosynthetic\_gene\_cluster | 32 | 25.8108108108 | 247 | 2e-64 |
| AAB66506.1 | tylactone\_synthase\_modules\_4\_&\_5 | BGC0000166 | Tylactone\_biosynthetic\_gene\_cluster | 29 | 30.7207207207 | 245 | 4.4e-64 |
| ABV83222.1 | CppJ | BGC0000116 | Nystatin-like\_Pseudonocardia\_polyene\_biosynthetic\_gene\_cluster | 34 | 23.5135135135 | 245 | 4.4e-64 |
| ABF92489.1 | mixed\_type\_I\_polyketide\_synthase\_-\_peptide\_synthetase | BGC0001025 | Myxovirescin\_biosynthetic\_gene\_cluster | 36 | 20.4954954955 | 245 | 7.6e-64 |
| ABC34154.1 | thiotemplate\_mechanism\_natural\_product\_synthetase | BGC0000961 | Bactobolin\_biosynthetic\_gene\_cluster | 32 | 24.954954955 | 244 | 9.9e-64 |
| AMB48441.1 | polyketide\_synthase | BGC0001357 | Carbamidocyclophane\_biosynthetic\_gene\_cluster | 33 | 23.018018018 | 244 | 1.3e-63 |
| AHE80994.1 | PieA4 | BGC0001169 | Piericidin\_A1\_biosynthetic\_gene\_cluster | 31 | 23.4684684685 | 244 | 1.3e-63 |
| AEU17897.1 | putative\_type\_I\_PKS | BGC0001072 | Simocyclinone\_biosynthetic\_gene\_cluster | 32 | 23.6036036036 | 243 | 1.7e-63 |
| CAN93351.1 | polyketide\_synthase | BGC0000179 | Etnangien\_biosynthetic\_gene\_cluster | 36 | 20.1351351351 | 242 | 4.9e-63 |
| WP\_010639241.1 | beta-ketoacyl\_synthase | BGC0000958 | Antimycin\_biosynthetic\_gene\_cluster | 36 | 20.1801801802 | 240 | 2.4e-62 |
| CAQ64687.1 | lasalocid\_modular\_polyketide\_synthase | BGC0000087 | Lasalocid\_biosynthetic\_gene\_cluster | 29 | 35.7657657658 | 239 | 3.2e-62 |
| BAG85027.1 | putative\_polyketide\_synthase | BGC0000086 | Lasalocid\_biosynthetic\_gene\_cluster | 29 | 35.7657657658 | 239 | 3.2e-62 |
| CAG23977.1 | polyketide\_synthase\_type\_I | BGC0000176 | Difficidin\_biosynthetic\_gene\_cluster | 30 | 25.045045045 | 238 | 5.4e-62 |
| AKA54627.1 | PKS | BGC0001216 | Splenocin\_biosynthetic\_gene\_cluster | 36 | 20.1801801802 | 238 | 5.4e-62 |
| AFL48528.1 | laidlomycin\_polyketide\_synthase\_(module\_7\_and\_module\_8) | BGC0000084 | Laidlomycin\_biosynthetic\_gene\_cluster | 29 | 27.972972973 | 238 | 7.1e-62 |
| AAC46026.1 | polyketide\_synthase\_modules\_4\_and\_5 | BGC0000113 | Niddamycin\_biosynthetic\_gene\_cluster | 29 | 31.6216216216 | 236 | 2.7e-61 |
| CAP95404.1 |  | BGC0001404 | Sorbicillin\_biosynthetic\_gene\_cluster | 33 | 23.963963964 | 236 | 2.7e-61 |
| ABB88521.1 | polyketide\_synthase\_type\_I | BGC0000050 | E-837\_biosynthetic\_gene\_cluster | 32 | 23.6036036036 | 235 | 4.6e-61 |
| AKQ22696.1 | malonyl\_CoA-acyl\_carrier\_protein\_transacylase | BGC0001186 | Misakinolide\_biosynthetic\_gene\_cluster | 32 | 24.5495495495 | 235 | 6e-61 |
| CAE52339.1 | Polyketide\_non-ribosomal\_peptide\_synthase | BGC0001088 | Albicidin\_biosynthetic\_gene\_cluster | 34 | 20.045045045 | 234 | 1e-60 |
| AAZ77698.1 | ChlA5 | BGC0000036 | Chlorothricin\_biosynthetic\_gene\_cluster | 30 | 26.8918918919 | 234 | 1e-60 |
| AFV96142.1 | polyketide\_synthase | BGC0001064 | Cylindrocyclophane\_biosynthetic\_gene\_cluster | 31 | 24.954954955 | 234 | 1e-60 |
| AFX60318.1 | polyketide\_synthase | BGC0001031 | Oocydin\_A\_biosynthetic\_gene\_cluster | 29 | 24.7747747748 | 233 | 3e-60 |
| AAO65800.1 | monensin\_polyketide\_synthase\_modules\_7\_and\_8 | BGC0000100 | Monensin\_biosynthetic\_gene\_cluster | 30 | 25.7207207207 | 233 | 3e-60 |
| CCP20050.1 | divL3\_protein | BGC0001119 | Divergolide\_biosynthetic\_gene\_cluster | 27 | 35.6756756757 | 233 | 3e-60 |
| AEZ53952.1 | polyketide\_synthase | BGC0000144 | Salinomycin\_biosynthetic\_gene\_cluster | 30 | 29.5045045045 | 232 | 6.6e-60 |
| AFX60311.1 | polyketide\_synthase | BGC0001031 | Oocydin\_A\_biosynthetic\_gene\_cluster | 33 | 20.5855855856 | 229 | 3.3e-59 |
| ABC35796.1 | polyketide\_synthase,\_putative | BGC0001102 | Malleilactone\_biosynthetic\_gene\_cluster | 31 | 24.0990990991 | 229 | 3.3e-59 |
| AEK75503.1 | type\_1\_polyketide\_synthase | BGC0000001 | Abyssomicin\_biosynthetic\_gene\_cluster | 31 | 24.5945945946 | 229 | 4.3e-59 |
| AFX60341.1 | polyketide\_synthase | BGC0001032 | Oocydin\_A\_biosynthetic\_gene\_cluster | 29 | 24.7297297297 | 228 | 5.6e-59 |
| AAO65798.1 | monensin\_polyketide\_synthase\_modules\_3\_and\_4 | BGC0000100 | Monensin\_biosynthetic\_gene\_cluster | 31 | 25.5855855856 | 228 | 9.6e-59 |
| AHD05615.1 | putative\_non-ribosomal\_peptide\_ligase/\_polyketide\_synthase\_hybrid | BGC0001033 | Paenilamicin\_biosynthetic\_gene\_cluster | 34 | 20.4504504505 | 227 | 2.1e-58 |
| BAQ25482.1 | type\_I\_polyketide\_synthase | BGC0001288 | Maklamicin\_biosynthetic\_gene\_cluster | 30 | 27.7027027027 | 226 | 2.8e-58 |
| AEC04356.1 | polyketide\_synthase | BGC0000178 | Elansolid\_biosynthetic\_gene\_cluster | 32 | 23.3333333333 | 225 | 6.2e-58 |
| AHB82062.1 | polyketide\_synthase | BGC0001231 | Microsclerodermins\_biosynthetic\_gene\_cluster | 34 | 20.3153153153 | 223 | 2.4e-57 |
| BAB69199.1 | modular\_polyketide\_synthase | BGC0000117 | Oligomycin\_biosynthetic\_gene\_cluster | 31 | 26.5315315315 | 223 | 2.4e-57 |
| ABC84457.1 | NigAII | BGC0000114 | Nigericin\_biosynthetic\_gene\_cluster | 30 | 28.2882882883 | 221 | 9e-57 |
| CDM36726.1 | Beta-ketoacyl\_synthase | BGC0001360 | Mycophenolic\_acid\_biosynthetic\_gene\_cluster | 33 | 23.6036036036 | 220 | 1.5e-56 |
| AAS92537.1 | PKS1 | BGC0001044 | Sirodesmin\_biosynthetic\_gene\_cluster | 33 | 20.6306306306 | 220 | 2e-56 |
| AAO65797.1 | monensin\_polyketide\_synthase\_module\_2 | BGC0000100 | Monensin\_biosynthetic\_gene\_cluster | 28 | 27.9279279279 | 220 | 2.6e-56 |
| CBK62731.1 |  | BGC0001115 | Thiomarinol\_biosynthetic\_gene\_cluster | 32 | 20.4504504505 | 218 | 5.8e-56 |
| AAM12934.1 | MmpF | BGC0000182 | Mupirocin\_biosynthetic\_gene\_cluster | 32 | 20.6306306306 | 218 | 7.6e-56 |
| BAD22832.1 | polyketide\_synthase | BGC0001265 | Melanin\_biosynthetic\_gene\_cluster | 31 | 23.5135135135 | 213 | 3.2e-54 |
| EAA59563.1 | hypothetical\_protein | BGC0000057 | F9775\_biosynthetic\_gene\_cluster | 33 | 20.1351351351 | 211 | 1.2e-53 |
| ABM63529.1 | BryX | BGC0000174 | Bryostatin\_biosynthetic\_gene\_cluster | 31 | 20.7207207207 | 207 | 1.3e-52 |
| AHB82051.1 | polyketide\_synthase | BGC0001019 | Microsclerodermins\_biosynthetic\_gene\_cluster | 32 | 20.1351351351 | 206 | 3e-52 |
| ADM79459.1 | PKS16\_protein | BGC0001266 | Grayanic\_acid\_biosynthetic\_gene\_cluster | 31 | 20.7657657658 | 204 | 1.1e-51 |
| AAN59953.1 | polyketide\_synthase\_1 | BGC0001258 | 1,8-dihydroxynaphthalene\_biosynthetic\_gene\_cluster | 32 | 20.1351351351 | 204 | 1.5e-51 |
| CAG23961.2 | polyketide\_synthase | BGC0001089 | Bacillaene\_biosynthetic\_gene\_cluster | 29 | 21.6666666667 | 203 | 3.3e-51 |
| AFR69335.1 | polyketide\_synthase\_SpiC2 | BGC0001045 | Spiruchostatin\_biosynthetic\_gene\_cluster | 33 | 20.1801801802 | 200 | 2.8e-50 |
| AAT69682.1 | polyketide\_synthase | BGC0001247 | Cercosporin\_biosynthetic\_gene\_cluster | 29 | 20.7207207207 | 195 | 5.3e-49 |
| EAQ86392.1 | hypothetical\_protein | BGC0001405 | Chaetoviridin\_/\_chaetomugilin\_biosynthetic\_gene\_cluster | 32 | 20.1351351351 | 195 | 9e-49 |
| AME18003.1 | enediyne\_polyketie\_synthase | BGC0001378 | Tiancimycin\_biosynthetic\_gene\_cluster | 26 | 33.1081081081 | 186 | 3.2e-46 |
| AHD05619.1 | putative\_polyketide\_synthase\_subunit | BGC0001033 | Paenilamicin\_biosynthetic\_gene\_cluster | 32 | 20.1801801802 | 181 | 1.3e-44 |
| AMK92560.1 | enediyne\_polyketide\_synthase | BGC0001377 | Uncialamycin\_biosynthetic\_gene\_cluster | 26 | 32.8378378378 | 172 | 4.8e-42 |
| CAJ76285.1 | putative\_polyketide\_synthase | BGC0000972 | Colibactin\_biosynthetic\_gene\_cluster | 31 | 21.5765765766 | 171 | 1.1e-41 |
| ABV97155.1 | Acyl\_transferase | BGC0000137 | Rifamycin\_biosynthetic\_gene\_cluster | 27 | 20.2252252252 | 145 | 6.2e-34 |
| ABP55220.1 | beta-ketoacyl\_synthase | BGC0000142 | Salinilactam\_biosynthetic\_gene\_cluster | 27 | 20.6306306306 | 144 | 1.1e-33 |
| ADN68484.1 | SorI | BGC0000184 | Sorangicin\_biosynthetic\_gene\_cluster | 26 | 24.0990990991 | 143 | 4e-33 |
| BAQ25481.1 | type\_I\_polyketide\_synthase | BGC0001288 | Maklamicin\_biosynthetic\_gene\_cluster | 27 | 20.3153153153 | 142 | 5.3e-33 |
| XP\_011392698.1 | hypothetical\_protein | BGC0001281 | Ustilagic\_acid\_biosynthetic\_gene\_cluster | 23 | 25.3603603604 | 142 | 5.3e-33 |
| ACN69989.1 | polyketide\_synthase | BGC0000079 | Indanomycin\_biosynthetic\_gene\_cluster | 25 | 28.8738738739 | 136 | 4.9e-31 |
| AGI61676.1 | modular\_PKS | BGC0000095 | Micromonolactam\_biosynthetic\_gene\_cluster | 25 | 27.8828828829 | 134 | 1.4e-30 |
| BAQ25513.1 | type\_I\_polyketide\_synthase | BGC0001288 | Maklamicin\_biosynthetic\_gene\_cluster | 27 | 20.6306306306 | 132 | 7.1e-30 |
| ABC87512.1 | polyketide\_synthase | BGC0001011 | Meridamycin\_biosynthetic\_gene\_cluster | 26 | 23.3333333333 | 129 | 4.6e-29 |
| ACY06289.1 | type\_I\_polyketide\_synthase | BGC0001042 | Sanglifehrin\_A\_biosynthetic\_gene\_cluster | 28 | 22.9279279279 | 128 | 1.3e-28 |
| CAQ52623.1 | type\_I\_polyketide\_synthase,\_module\_6 | BGC0001066 | Kendomycin\_biosynthetic\_gene\_cluster | 26 | 24.3693693694 | 126 | 3.9e-28 |
| BI06\_RS32440 | polyketide\_synthase | BGC0001232\_c2 | Leucanicidin\_biosynthetic\_gene\_cluster | 27 | 20.2252252252 | 124 | 1.9e-27 |
| ADM46359.1 | polyketide\_synthase | BGC0000106 | Naphthomycin\_biosynthetic\_gene\_cluster | 25 | 30.3153153153 | 122 | 5.7e-27 |
| ADM46360.1 | polyketide\_synthase | BGC0000106 | Naphthomycin\_biosynthetic\_gene\_cluster | 27 | 20.3153153153 | 115 | 9e-25 |
| CCP20048.1 | divL1\_protein | BGC0001119 | Divergolide\_biosynthetic\_gene\_cluster | 25 | 22.8378378378 | 110 | 2.9e-23 |
